# Living on a farm, contact with farm animals and pets, and childhood acute lymphoblastic leukemia: pooled and meta‐analyses from the Childhood Leukemia International Consortium

**DOI:** 10.1002/cam4.1466

**Published:** 2018-04-16

**Authors:** Laurent Orsi, Corrado Magnani, Eleni T. Petridou, John D. Dockerty, Catherine Metayer, Elizabeth Milne, Helen D. Bailey, Nick Dessypris, Alice Y. Kang, Catharina Wesseling, Claire Infante‐Rivard, Victor Wünsch‐Filho, Ana M. Mora, Logan G. Spector, Jacqueline Clavel

**Affiliations:** ^1^ INSERM U1153, Epidemiology and Biostatistics Sorbonne Paris Cité Center (CRESS) Epidemiology of Childhood and Adolescent Cancers Team (EPICEA) Paris‐Descartes University Villejuif France; ^2^ Dipartimento di Medicina Traslazionale Università del Piemonte Orientale AOUMaggiore della Carità & CPO Piemonte Novara Italy; ^3^ Department of Hygiene, Epidemiology, and Medical Statistics School of Medicine University of Athens Athens Greece; ^4^ Department of Medicine Clinical Epidemiology Unit Karolinska Institute Stockholm Sweden; ^5^ Department of Preventive and Social Medicine Dunedin School of Medicine University of Otago Dunedin New Zealand; ^6^ School of Public Health University of California Berkeley California; ^7^ Telethon Kids Institute University of Western Australia Perth Western Australia Australia; ^8^ Unit of Occupational Medicine Institute of Environmental Medicine Karolinska Institutet Stockholm Sweden; ^9^ Department of Epidemiology, Biostatistics, and Occupational Health Faculty of Medicine McGill University Montréal Quebec Canada; ^10^ School of Public Health University of Saõ Paulo Saõ Paulo Brazil; ^11^ Central American Institute for Studies on Toxic Substances (IRET) Universidad Nacional Heredia Costa Rica; ^12^ Division of Epidemiology and Clinical Research Department of Pediatrics and Masonic Cancer Center University of Minnesota Minneapolis Minnesota

**Keywords:** Animals, childhood leukemia, contact, farm residence

## Abstract

The associations between childhood acute lymphoblastic leukemia (ALL) and several factors related to early stimulation of the immune system, that is, farm residence and regular contacts with farm animals (livestock, poultry) or pets in early childhood, were investigated using data from 13 case–control studies participating in the Childhood Leukemia International Consortium. The sample included 7847 ALL cases and 11,667 controls aged 1–14 years. In all studies, the data were obtained from case and control parents using standardized questionnaires. Pooled odds ratios (ORs) and 95% confidence intervals (CIs) were estimated by unconditional logistic regression adjusted for age, sex, study, maternal education, and maternal age. Contact with livestock in the first year of life was inversely associated with ALL (OR = 0.65, 95% CI: 0.50, 0.85). Inverse associations were also observed for contact with dogs (OR = 0.92, 95% CI: 0.86, 0.99) and cats (OR = 0.87, 95% CI: 0.80, 0.94) in the first year of life. There was no evidence of a significant association with farm residence in the first year of life. The findings of these large pooled and meta‐analyses add additional evidence to the hypothesis that regular contact with animals in early childhood is inversely associated with childhood ALL occurrence which is consistent with Greaves’ delayed infection hypothesis.

## Introduction

Acute leukemia (AL) is the most common cancer in children under 15 years of age 1, 2, 3, 4. Numerous studies have focused on childhood acute lymphoblastic leukemia (ALL) the most frequent type of AL. Birth characteristics, chemical exposures, and various surrogates of priming of the immune system have been identified as risk factors of childhood leukemia 5.

According to Greaves’ delayed infection hypothesis 6, lack of immune stimulation during early childhood could lead to ALL occurrence later in childhood. Support for this hypothesis comes from epidemiological studies which have found evidence of significant protective associations between ALL and several proxies of early immune stimulation including early daycare attendance 7 and breastfeeding for more than 6 months 8, 9. Further support also comes from the recent pooled analysis 10 conducted within the framework of the Childhood Leukemia International Consortium (CLIC) 11 which showed a significant inverse association between early daycare attendance and ALL, with an inverse trend with lower age at first daycare attendance. In this same pooled study, prolonged breastfeeding was also significantly inversely linked to the risk of ALL.

An additional early source of immune stimulation may be regular contacts with animals in early childhood, as has been hypothesized for allergies 12, 13. However, the childhood ALL literature on this topic is still limited. Discordant findings have been reported from five case–control studies 14, 15, 16, 17, 18 on childhood ALL and contact with animals, using different definitions of exposure and different time windows of interest. The French case–control study ESTELLE 18 found significant inverse associations with early regular contact with cattle (odds ratio (OR) = 0.3, 95% confidence interval (CI): 0.2, 0.7), cats (OR = 0.7, 95% CI: 0.6, 1.0), and all pets combined (OR = 0.8, 95% CI: 0.7, 1.0), consistent with previous French findings 17 which had not reached statistical significance. By contrast, two studies 14, 15 found increased ORs with exposure to cats and any pets during childhood while the fifth study, conducted in the USA and Canada, found no association between ALL and dog or cat ownership (OR = 1.0, 95% CI: 0.9, 1.2; OR = 0.9, 95% CI: 0.8, 1.1) from preconception to diagnosis 16.

Farm residence involves exposure to animals, especially farm animals, and to various organic dusts, toxins, and pesticides. The limited literature (three case–control studies) 17, 18, 19 suggests a decreased risk of childhood ALL with farm residence in the first year of life 17, any time during childhood 19 or with frequent farm visits during the first year of life 18.

In this study, we used a unique large set of case–control studies from CLIC to examine whether living on a farm or regular contact with livestock, poultry, and pets in early childhood (which we defined as the first year of life) was inversely associated with childhood ALL.

## Materials and Methods

The data for this analyses were provided by principal investigators of 13 CLIC case–control studies conducted in nine countries between 1980 and 2013 (Table 1). The following studies were included in the current analysis: Australian Study of Causes of Acute Lymphoblastic Leukemia in Children (AUS_ALL) 20; State of Sao Paulo Childhood acute lymphoblastic leukemia study, Brazil (BRA_SAOP) 21; Quebec Childhood Leukemia Study, Canada (CA_QCLS) 22; Costa Rican Childhood Leukemia Study (CR_CRCLS) 23; Adele Study, France (FR_ADELE) 24; Electre Study, France (FR_ELECTRE) 25; Epidemiologic Study on Childhood Cancer and Leukemia, France (FR_ESCALE) 17; Epidemiologic Study on Childhood Cancer, Leukemia and lymphoma, France (FR_ESTELLE) 18; Nationwide Registration for Childhood Hematological Malignancies, Greece (GR_NARECHEM) 26; Study on the Etiology of Childhood Lymphohematopoietic Malignancies, Italy (IT_SETIL) 27; New Zealand Childhood Cancer Study (NZ_NZCCS) 19; Children's Oncology Group Study, United States (US_COG15) 16; and Northern California Childhood Leukemia Study, United States (US_NCCLS) 28.

**Table 1:** Summary of the 13 studies included in the pooled analysis (1980–2013), and numbers of cases of acute lymphoblastic leukemia and controls aged 1 to 14 years old, Childhood Leukemia International Consortium

Country, Study (Reference no.) (period^a^)	ALL cases			Controls			Data collection method	Exposure	Definition of exposure
	Source	Participation	*N*	Source	Participation	*N*			
Australia, AUS_ALL 20 (2002–2006)	Hospital (nationwide)	75%	379	Random digit dialing	64%	846	Self‐administered questionnaire	Farm residence	Amount of time living in a farm from birth to diagnosis >90%
Brazil (State of Saõ Paulo), BRA_SAOP 21 (2003–2009)		90%	152	Birth registry	88%	563	Face‐to‐face interview	Contact with animals	Animal ownership during the child's first year of life ‐ Pets: cats, dogs
Canada‐Québec, CA_QCLS 22 (1980–2000)	Hospital (nationwide)	93%	766	Health Insurance file population‐based registry (province‐wide)	86%	765	Telephone interview	Farm residence (only second part of the study 1994–2000)	Living on a farm during the child's first year of life
								Contact with animals	Animal ownership during the child's first year of life ‐ Pets: cats, dogs
Costa Rica, CR_CRCLS 23 (2001–2003)	Population‐based cancer registry and hospital (nationwide)	90%	241	Birth registry (nationwide)	90%	550	Face‐to‐face interview	Contact with animals	Regular contact with animal during the child's first year of life ‐ Pets: cats, dogs ‐ Livestock: cattle, pigs
France, FR_ADELE 24 (1994–1999)	Hospital	95%	232	Hospitals (same as cases)	99%	278	Face‐to‐face interview	Farm residence	Living on a farm during the child's first year of life
								Contact with animals	Animal ownership during the child's first year of life ‐ Pets: cats, dogs ‐ Livestock: cattle, pigs, sheep ‐ Poultry
France, FR_ELECTRE 25 (1995–1998)	Population‐based cancer registry (nationwide)	73%	401	Population quotas by age, sex, region (nationwide)	70%	532	Self‐administered questionnaire	Contact with animals	Animal ownership during the child's first year of life ‐ Pets: cat, dog ‐ Livestock: cattle, pigs, sheep ‐ Poultry
France, FR_ESCALE 17 (2003–2004)	Population‐based cancer registry (nationwide)	91%	635	Population quotas by age, sex, region (nationwide)	71%	1494	Telephone interview	Farm residence	Living on a farm during the child's first year of life
								Contact with animals	Regular contact with animal (at least once/week) during the child's first year of life ‐ Pets: cats, dogs ‐ Livestock: cattle, pigs, sheep ‐ Poultry
France, FR_ESTELLE 18 (2010–2011)	Population‐based cancer registry (nationwide)	93%	615	Population quotas by age, sex, region (nationwide)	86%	1225	Telephone interview	Farm residence	Living on a farm or frequent farm visit (>1 day/week) during the child's first year of life
								Contact with animals	Regular contact with animal (at least once/week) during the child's first year of life ‐ Pets: cats, dogs ‐ Livestock: cattle, pigs, sheep ‐ Poultry
Greece, GR_NARECHEM 26 (1996–2013)	Nationwide hospital cancer registry	83%	1087	Hospital, age and sex matched	96%	1214	Face‐to‐face interview	Farm residence	Living on a farm at reference date and no move since birth
								Contact with animals	Animal ownership during the child's first year of life ‐ Pets: cats, dogs ‐ Livestock: cattle, pigs, sheep ‐ Poultry
Italy, IT_SETIL 27 (1998–2001)	Nationwide clinical database	91%	585	Registry (nationwide)	70%	994	Face‐to‐face interview	Contact with animals	Animal ownership during the child's first year of life ‐ Pets: cats, dogs
New Zealand, NZ_NZCCS 19 (1990–1994)	Registry (nationwide)	92%	95	Birth registry (nationwide)	69%	116	Face‐to‐face interview	Farm residence	Living on a farm (duration >1 month) during the child's first year of life
								Contact with animals	Animal ownership during the child's first year of life ‐ Pets : cats, dogs ‐ Livestock: cattle, pigs, sheep (only for those residing in a farm) ‐ Poultry (only for those residing in a farm)
United States, US_COG15 16 (1989–1993)	Children's Cancer Group clinical trials	87%	1847	Random Digit Dialing	70%	1906	Telephone interview	Contact with animals	Animal ownership during the child's first year of life ‐ Pets: cats, dogs
United States (California), US_NCCLS 28 (1995–2008)	Hospitals	86%	812	Birth registry (statewide)	68%	1184	Face‐to‐face interview	Farm residence	Living on a farm during the child's first year of life
								Contact with animals	Regular contact with animal during the child's two‐first years of life ‐ Pets: cats, dogs
Total			7847			11,667			

ALL, acute lymphoblastic leukemia; AUS_ALL, Australian Study of Causes of Acute Lymphoblastic Leukemia in Children; BRA_SAOP, State of Sao Paulo Childhood acute lymphoblastic leukemia study (Brazil); CA_QCLS, Quebec Childhood Leukemia Study (Canada); CR_CRCLS, Costa Rican Childhood Leukemia Study (Costa Rica); FR_ADELE, Adele Study (France); FR_ELECTRE, Electre Study (France); FR_ESCALE, Epidemiologic Study on Childhood Cancer and Leukemia (France); FR_ESTELLE, Epidemiologic Study on Childhood Cancer, Leukemia and Lymphoma (France); GR_NARECHEM, Nationwide Registration for Childhood Hematological Malignancies (Greece); IT_SETIL, Study on the Etiology of Childhood Lymphohematopoietic Malignancies (Italy); NZ_NZCCS, New Zealand Childhood Cancer Study; US_COG15, Children's Oncology Group Study (United States); US_NCCLS, Northern California Childhood Leukemia Study (United States).

a Period refers to period of diagnosis for cases and period of recruitment for controls.

### Data collection

Study design and participant characteristics for each study have been described elsewhere [11.] Briefly, in all studies, data were obtained from case and control parents using standardized questionnaires which included details about socio‐demographic characteristics and information on factors potentially associated with AL. The data collection methods for each study are listed in Table 1.

For these present analyses, principal investigators of each study were asked to provide data on whether a child had ever lived on a farm (and if yes, when); whether the child had ever visited a farm and the timing and frequency of these visits; and whether the child had regular contact with animals or whether the family had owned any animals with details on time period and type of animal. Stratification variables (sex, age at diagnosis or recruitment) and parental characteristics (parental age at child's birth, parental education) were also requested, as well as any other variables used for matching and other indicators of socioeconomic status (SES; income, parental profession, ethnicity). For ALL cases, histological data were also provided for each study, except BRA_SAOP and CR_CRCLS, for which they were unavailable.

All of the studies were approved by institutional ethics committees.

### Data harmonization

#### Socio‐demographic characteristics

Age was defined as age at the reference date (diagnosis for cases and recruitment or questionnaire return for controls) and was categorized in eight classes (<2, 2, 3, 4, 5–6, 7–8, 9–11 and 12–14 years of age). Parental education levels were harmonized across all studies and categorized into three levels: none or primary education, secondary education, and college/university degree. Depending on the available data, a proxy for SES was derived from family annual income (AUS_ALL, CA_QCLS, US_COG15, US_NCCLS), parental professional category (BRA_SAOP, FR_ADELE, FR_ELECTRE, FR_ESCALE, FR_ESTELLE, GR_NARECHEM, NZ_NZCCS), parental education level (IT_SETIL), or housing characteristics (CR_CRCLS) and classed in three categories: low, medium, and high.

#### Farm residence

Data regarding farm residence in the first year of life were available in six studies (Table 1). Children whose parents reported that they lived on a farm at the child's year of birth (CA_QCLS, FR_ADELE, FR_ESCALE, NZ_NZCCS, US_NCCLS) or that their children had visited a farm at least twice a week in their first year of life (FR_ESTELLE) were classed as “living on a farm.” The Greek study (GR_NARECHEM) had data on the child's residence on a farm at the time of diagnosis and whether child had moved residence between birth and reference date, and we classed children who were living on a farm since birth as “living on a farm.” The Australian (AUS_ALL) study had data on cumulative time spent on a farm between birth and diagnosis, and we classed the children who had spent at least 90% of their life on a farm as “living on a farm.”

#### Contact with animals

##### Livestock

Contacts with livestock were defined as contacts with at least one type of livestock, (cattle, pigs, or sheep) in the first year of life, in their residence (FR_ADELE, FR_ELECTRE, GR_NARECHEM, NZ_NZCCS), or by regular visits (CR_CRCLS, FR_ESCALE, FR_ESTELLE). Seven studies (CR_CRCLS, FR_ADELE, FR_ELECTRE, FR_ESCALE, FR_ESTELLE, GR_NARECHEM, and NZ_NZCCS) included contacts with cattle and pigs and contacts with sheep were available in all of them except CR_CRCLS (Table 1). Binary exposure variables were generated for contact with any livestock and for contacts with each type of animal (cattle, pigs, sheep). A five‐class variable (no contact, contact with cattle only, contact with pigs only, contact with sheep only, contact with at least two livestock) was also created.

##### Poultry

Data on contact with poultry in the first year of life were available in six studies. A binary exposure variable was generated, by classing as exposed to poultry children whose parents had reported that they had poultry at their residence in the child's year of birth (FR_ADELE, FR_ELECTRE, GR_NARECHEM, NZ_NZCCS) or those who were reported to have had regular contact with poultry in their first year of life (FR_ESCALE, FR_ESTELLE).

##### Pets

For these analyses, a ‘pet’ was defined as either a cat or dog and 12 studies (BRA_SAOP, CA_QCLS, CR_CRCLS, FR_ADELE, FR_ELECTRE, FR_ESCALE, FR_ESTELLE, GR_NARECHEM, IT_SETIL, NZ_NZCCS, US_COG15, and US_NCCLS) had these data (Table 1). Binary variables were generated by types of pet, cats and dogs, and any pet. We classed as *exposed to pets* children whose parents had reported that they had a pet at their residence in the child's year of birth (BRA_SAOP, CA_QCLS, FR_ADELE, FR_ELECTRE, GR_NARECHEM, IT_SETIL, NZ_NZCCS, US_COG15) or those who were reported to have had regular contact with pets in their first year of life (CR_CRCLS, FR_ESCALE, FR_ESTELLE) or in their two‐first years of life (US_NCCLS). For the later study (US_NCCLS), as only data on regular contact with pets in the two‐first years of life were available, we hypothesized that this period would be a good surrogate for exposures occurring in the first year of life. A four‐class variable (no contact, contact with dogs only, contact with cats only, contact with both dogs and cats) was also created to take into account the different combinations of pet contact.

### Statistical analysis

The individual participant data (IPD) were analyzed using SAS v9.3 (SAS Institute, Cary NC) and R v3.3.1 software. We restricted the analyses to children aged at least 1 year of age, in order to ensure that the ALL cases and controls had the opportunity to have lived or visited a farm or to be exposed to animals during their first year of life. The analyses were performed for overall ALL and by ALL subtypes, B lineage and T lineage ALL. Analyses were also stratified by age (2–5, 6 to 14 years of age) and sex.

#### Meta‐analysis

Study‐specific ORs were estimated for the main exposures of interest and summarized using IPD meta‐analyses with a two‐stage procedure. First, the study‐specific OR and 95% CIs were estimated, using either unconditional or conditional logistic regression, depending on the original study design, and including study‐specific matching variables in the models. The socio‐demographic characteristics significantly associated with both the case–control status and exposure were also included in the study‐specific models. We investigated between‐study heterogeneity by calculating Cochran's *Q* 29 and *I* ^2^ statistics 30. Then, summary ORs and 95% CI were estimated using either the fixed‐effect model or, if the *I* ^2^ statistic was greater than 0, the random‐effect model 31, regardless of the conclusion of the Cochran's test. Forest plots of study‐specific ORs and summary‐statistics were produced.

#### Pooled analysis

Pooled ORs and their 95% CIs were estimated from the pooled individual data using unconditional logistic regression adjusted for age, sex, and a categorical variable denoting study of origin. Maternal education and maternal age at the child's birth were also included in the models, because they were significantly associated with both case–control status and exposure. We assessed two‐way interaction terms between exposure variables (i.e., living on a farm, contact with livestock, poultry, and pets) and conducted stratified analyses to determine whether the effect estimates of a single exposure differed by strata of another exposure.

#### Sensitivity analysis and additional adjustments (pooled analysis)

Analyses were repeated using a one‐stage meta‐analysis procedure. For each exposure of interest, ORs and their 95% CIs were estimated from the pooled individual data using unconditional logistic regression adjusted for age, maternal education, and maternal age at the child's birth. The models also included a random effect for the intercept and a random slope for the exposure of interest.

Robustness of the results was assessed by excluding one study at a time from the pooled analyses. To detect potential selection bias related to study design, we also repeated the analyses with hospital‐based case–control studies excluded (i.e., FR_ADELE, GR_NARECHEM) from the analyses. Analyses were also repeated adjusting for SES instead of maternal education and adjusting for daycare attendance and breastfeeding during the first year of life (yes/no), both factors associated with ALL in previous pooled analyses 10. Maternal home pesticide use and preconception paternal smoking, which are potential risk factors for childhood ALL 32, 33, were not available for all individual studies. Thus, to assess the impact of those unmeasured confounders, we performed deterministic sensitivity analyses using the Episens procedure 34 in STATA v11.2 (StataCorp LP, College Station, TX, 2009). The method allows the taking into account of an unmeasured confounder (K) in the association between ALL (D) and the exposure of interest (E), by fixing three parameters: the magnitude of the association between D and K in terms of OR and the prevalence of the exposure to K among the subjects exposed to E and among those unexposed to E. Back‐calculation using this set of parameters gives an estimate for the OR between D and E adjusted for the unobserved confounder K.

For each exposure of interest, the potential for participation bias was also assessed by estimating the difference in participation between exposed and unexposed controls that would have generated an OR of the observed magnitude, under the assumption of no true association.

The 95% CI and two‐sided *P*‐values were calculated, even though the question was one‐sided.

## Results

Overall, data from 13 studies with 7847 ALL cases (B lineage ALL 76%, T lineage ALL 10%, 8% other or unspecified ALL and missing histological type 5%) and 11,667 controls were included in the analyses (Table 2). The cases were slightly younger than the controls (5.2 years vs. 5.6 years) and more likely to be boys.

**Table 2:** Distribution of age, sex and socioeconomic characteristics by case/control status, children aged 1 to 14 years old, pooled analysis of 13 studies (1980–2013), Childhood Leukemia International Consortium

	ALL (*n* = 7847)		Controls (*n* = 11,667)		OR^a^	95% CI	*P*
	*n*	%	*n*	%			
Histological type^b^							
B lineage ALL	5967	76					
BCP ALL	5837	74					
T lineage ALL	820	10					
Mixed lineage ALL	19	0.2					
Unspecified ALL	648	8					
Missing	393	5					
Child's age (years)							<0.0001
Mean (SD)	5.2 (3.5)		5.6 (3.6)				
<2	648	8	1106	9	0.76	0.67, 0.85	
2	1323	17	1660	14	1.00	Ref	
3	1332	17	1699	15	0.98	0.89, 1.09	
4	1030	13	1457	13	0.90	0.81, 1.01	
5–6	1319	17	2011	17	0.86	0.77, 0.95	
7–8	780	10	1278	11	0.81	0.72, 0.91	
9–11	782	10	1266	11	0.80	0.71, 0.90	
12–14	633	8	1190	10	0.69	0.61, 0.79	
Child's sex							0.07
Girl	3414	44	5260	45	1.00	Ref	
Boy	4433	56	6407	55	1.06	0.99, 1.12	
Maternal age (years)							<0.0001
<25	2028	26	2505	22	1.20	1.11, 1.29	
25–29	2638	34	4028	35	1.00	Ref	
30–34	2147	27	3306	29	1.02	0.95, 1.10	
≥35	1009	13	1684	14	0.98	0.89, 1.08	
Missing	25		144				
Maternal education (highest degree)							<0.0001
Did not complete secondary education	1873	24	2881	25	1.25	1.15, 1.36	
Completed secondary education	3437	44	4725	41	1.00	Ref	
Completed tertiary education	2508	32	3947	34	0.98	0.91, 1.05	
Missing	29		114				
Socioeconomic status							<0.0001
Low	1916	25	2340	21	1.20	1.11, 1.30	
Medium	3261	42	4889	43	1.00	Ref	
High	2529	33	4126	36	0.93	0.86, 1.00	
No occupation or missing	141		312				

ALL, acute lymphoblastic leukemia; BCP ALL, B‐cell precursor acute lymphoblastic leukemia; BRA_SAOP, State of Sao Paulo Childhood Acute Lymphoblastic Leukemia Study (Brazil); CI, confidence interval; CR_CRCLS, Costa Rican Childhood Leukemia Study (Costa Rica); OR, odds ratio.

a Odds ratio and 95% confidence interval were estimated by unconditional logistic regression adjusted for child's age at reference date, child's sex, and study of origin.

b Data on ALL histological type were not available for BRA_SAOP (*n* = 152) and CR_CRCLS (*n* = 241).

### Socio‐demographic characteristics

Case mothers were more often less than 25 years of age at the index child's birth (OR = 1.20, 95% CI: 1.11, 1.29) and less educated than control mothers (OR = 1.25, 95% CI: 1.15, 1.36). Case parents also tended to be in the lowest SES category compared to control parents (OR = 1.20, 95% CI: 1.11, 1.30; Table 2).

Controls whose parents were in the highest SES category or whose mother had the highest educational level were reported to have lived less often on a farm or to have had less frequent contact with livestock in the first year of life (Table S1). By contrast, controls whose parents were in the highest SES category or whose mothers had the highest educational level were reported to have had more frequent contact with pets in the first year of life. Finally, early contact with livestock was more common among controls who had lived on a farm in their first year of life and associated with regular contact with pets.

### Living on a farm

**Figure 1:**
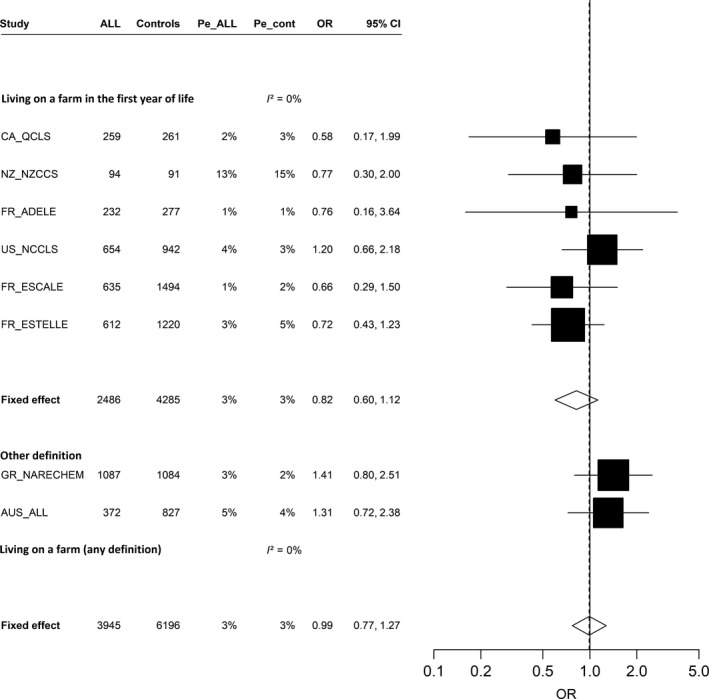
Association between acute lymphoblastic leukemia and living on a farm (yes vs. no), (restricted to children aged ≥1 year), meta‐analysis of eight studies (1980–2013), Childhood Leukemia International Consortium. ALL, acute lymphoblastic leukemia; AUS_ALL, Australian Study of Causes of Acute Lymphoblastic Leukemia in Children; CA_QCLS, Quebec Childhood Leukemia Study (Canada); CI, confidence interval; FR_ADELE, Adele Study (France); FR_ESCALE, Epidemiological Study on Childhood Cancer and Leukemia (France); FR_ESTELLE, Epidemiologic Study on Childhood Cancer, Leukemia and lymphoma (France); GR_NARECHEM, Nationwide Registration for Childhood Hematological Malignancies (Greece); NZ_NZCCS, New Zealand Childhood Cancer Study (New Zealand); OR, odds ratio; Pe_ALL, prevalence of exposure among acute lymphoblastic leukemia cases; Pe_cont, prevalence of exposure among controls; US_NCCLS, Northern California Childhood Leukemia Study (US). Studies are ordered by increasing study period. Study‐specific odds ratios and 95% confidence interval were estimated by conditional (CA_QCLS, GR_NARECHEM, NZ_NZCCS, US_NCCLS) or unconditional (AUS_ALL, FR_ADELE, FR_ESCALE, FR_ESTELLE) logistic models, adjusted for child's age at reference date, sex, maternal educational level (AUS_ALL, GR_NARECHEM, NZ_NZCCS, US_NCCLS), ethnicity (FR_ADELE, NZ_NZCCS), region or center of recruitment (FR_ADELE), region or state of residence (AUS_ALL), “urban/rural” status of the place of residence (GR_NARECHEM, FR_ESTELLE), parental professional category (FR_ESCALE, FR_ESTELLE, GR_NARECHEM), household income (US_NCCLS), maternal age at child's birth (AUS_ALL, CA_QCLS, FR_ESCALE, FR_ESTELLE, US_NCCLS).

Living on a farm overall was not associated with ALL (pooled OR = 1.09, 95% CI: 0.86, 1.36; [Table 3]; meta‐OR = 0.99, 95% CI: 0.77, 1.27; [Fig. 1]). No association was found with having lived on a farm or having had regular farm visits before 1 year old (pooled OR = 0.93, 95% CI: 0.70, 1.24) from the six studies with these data, with few exposed children (3% of both cases and controls). Similar results were observed in the meta‐analysis (meta‐OR = 0.82, 95% CI: 0.60, 1.12), and no heterogeneity was seen (Fig. 1).

**Table 3:** Association between acute lymphoblastic leukemia, farm residence, and contact with livestock and poultry in the first year of life, pooled analysis of 10 studies (1980–2013), children aged 1 to 14 years old, Childhood Leukemia International Consortium

	Number of studies	ALL		Controls		OR^a^	95% CI	P
		*n*	%	*n*	%			
Living on a farm (any definitions)	8^b^							
No		3898	97	6343	97	1.00	Ref.	
Yes		134	3	198	3	1.09	0.86, 1.36	0.48
Missing		113		105				
Living on a farm in the first year of life	6^c^							
No		2494	97	4361	97	1.00	Ref.	* *
Yes		79	3	140	3	0.93	0.70, 1.24	0.64
Missing		106		85				
Contact with any livestock in the first year of life	7^d^							
No		3193	98	5155	96	1.00	Ref.	
Yes		79	2	219	4	0.65	0.50, 0.85	0.002
Missing		34		35				
Contact with cattle	7^d^							
No		3235	99	5227	97	1.00	Ref.	
Yes		44	1	155	3	0.54	0.39, 0.77	0.0006
Missing		27		27				
Contact with pigs	7^d^							
No		3260	99	5316	99	1.00	Ref.	
Yes		19	1	66	1	0.58	0.35, 0.98	0.04
Missing		27		27				
Contact with sheep	6^e^							
No		2996	99	4731	98	1.00	Ref.	
Yes		44	1	104	2	0.68	0.47, 0.98	0.04
Missing		25		24				
Contact with any livestock in the first year of life	6^e^							
No		2968	98	4645	96	1.00	Ref.	0.02
Contact with cattle only		18	0.6	58	1.2	0.59	0.34, 1.01	
Contact with pigs only		3	0.1	7	0.1	0.95	0.24, 3.74	
Contact with sheep only		29	0.9	47	1.0	0.91	0.56, 1.46	
Contact with at least two types of animals		18	0.6	71	1.5	0.44	0.26, 0.75	
Missing		29		31				
Contact with poultry in the first year of life	6^e^							
No		2926	97	4584	95	1.00	Ref.	
Yes		105	3	242	5	0.78	0.62, 1.00	0.05
Missing		34		33				

ALL, acute lymphoblastic leukemia; AUS_ALL, Australian Study of Causes of Acute Lymphoblastic Leukemia in Children; CA_QCLS, Quebec Childhood Leukemia Study (Canada); CI, confidence interval; CR_CRCLS, Costa Rican Childhood Leukemia Study (Costa Rica); FR_ADELE, Adele Study (France); FR_ELECTRE, Electre Study (France); FR_ESCALE, Epidemiologic Study on Childhood Cancer and Leukemia (France); FR_ESTELLE, Epidemiologic Study on Childhood Cancer, Leukemia and lymphoma (France); GR_NARECHEM, Nationwide Registration for Childhood Hematological Malignancies (Greece); NZ_NZCCS, New Zealand Childhood Cancer Study (New Zealand); OR, odds ratio; US_NCCLS, Northern California Childhood Leukemia Study (United States).

a Odds ratio and 95% confidence interval were estimated by unconditional logistic regression adjusted for child's age at reference date, child's sex, maternal age at child's birth, maternal educational level, and study of origin.

b Eight studies (n = 4145 cases, n = 6646 controls): AUS_ALL, CA_QCLS, FR_ADELE, FR_ESCALE, FR_ESTELLE, GR_NARECHEM, NZ_NZCCS, US_NCCLS.

c Six studies (n = 2679 cases, n = 4586 controls): CA_QCLS, FR_ADELE, FR_ESCALE, FR_ESTELLE, NZ_NZCCS, US_NCCLS.

d Seven studies (n = 3306 cases, n = 5409 controls): CR_CRCLS, FR_ADELE, FR_ELECTRE, FR_ESCALE, FR_ESTELLE, GR_NARECHEM, NZ_NZCCS.

e Six studies (n = 3065 cases, n = 4859 controls): FR_ADELE, FR_ELECTRE, FR_ESCALE, FR_ESTELLE, GR_NARECHEM, NZ_NZCCS.

### Contacts with livestock and poultry

**Figure 2:**
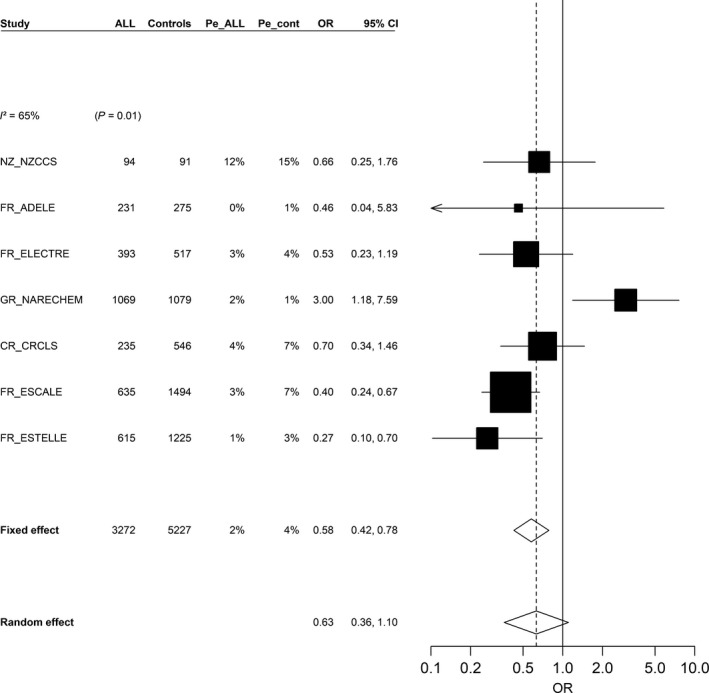
Association between acute lymphoblastic leukemia and contact with livestock in the first year of life (yes vs. no), (restricted to children aged ≥1 year), meta‐analysis of seven studies (1990–2013), Childhood Leukemia International Consortium. ALL, acute lymphoblastic leukemia; CI, confidence interval; CR_CRLS, Costa Rican Childhood Leukemia Study (Costa Rica); FR_ADELE, Adele Study (France); FR_ELECTRE, Electre Study (France); FR_ESCALE, Epidemiological Study on Childhood Cancer and Leukemia (France); FR_ESTELLE, Epidemiologic Study on Childhood Cancer, Leukemia and Lymphoma (France); GR_NARECHEM, Nationwide Registration for Childhood Hematological Malignancies (Greece); NZ_NZCCS, New Zealand Childhood Cancer Study (New Zealand); OR, odds ratio; Pe_ALL, prevalence of exposure among acute lymphoblastic leukemia cases; Pe_cont, prevalence of exposure among controls. Studies are ordered by increasing study period. Study‐specific odds ratios and 95% confidence interval were estimated by conditional (GR_NARECHEM, NZ_NZCCS) or unconditional (CR_CRLS, FR_ADELE, FR_ELECTRE, FR_ESCALE, FR_ESTELLE) logistic models, adjusted for child's age at reference date, sex, maternal educational level (FR_ELECTRE, GR_NARECHEM, NZ_NZCCS), ethnicity (FR_ADELE, NZ_NZCCS), region or center of recruitment (FR_ADELE), region or state of residence (FR_ELECTRE), “urban/rural” status of the place of residence (GR_NARECHEM, FR_ESTELLE), parental professional category (CR_CRLS, FR_ELECTRE, FR_ESCALE, FR_ESTELLE, GR_NARECHEM), maternal age at child's birth (FR_ELECTRE, FR_ESCALE, FR_ESTELLE).

Using data from seven studies, early contact with livestock was inversely associated with ALL (pooled OR = 0.65, 95% CI: 0.50, 0.85; Table 3). Six individual studies showed inverse associations, with ORs ranging from 0.27 to 0.70, while the Greek study had a significant positive association, although based on imprecise estimates (OR = 3.00, 95% CI: 1.18, 7.59; Fig. 2). The meta‐OR was close to that found in the pooled analyses (OR = 0.63, 95% CI: 0.36, 1.10), but there was high between‐study heterogeneity (I²=65%, *P = 0.01*; Fig. 2). In the pooled analysis, significant inverse associations were seen with contact with cattle (OR = 0.54, 95% CI: 0.39, 0.77), pigs (OR = 0.58, 95% CI: 0.35, 0.98), and sheep (OR = 0.68, 95% CI: 0.47, 0.98) in the first year of life. Of the 251 children exposed to livestock, 162 (65%) were exposed to only one type of animal and 89 (35%) have ever had early contact with more than one type of animal. Contact with cattle was inversely associated with ALL, either alone (OR = 0.59, 95% CI: 0.34, 1.01) or with any other types of animal (OR = 0.44, 95% CI: 0.26, 0.75), whereas contacts with pigs or sheep alone were not associated with ALL. Contact with poultry was also inversely associated with ALL (OR = 0.78, 95% CI: 0.62 to 1.00).

Similar estimates were observed in the meta‐analyses with significant associations with contacts with cattle and pigs (OR = 0.53, 95% CI: 0.33, 0.84; OR = 0.58, 95% CI: 0.34, 1.00; respectively), but there was high between‐study heterogeneity for contacts with sheep (*I* ^2^ = 64%, *P = 0.02*; Figs. [Link], [Link], [Link]). Between‐study heterogeneity was also observed for contact with poultry (*I* ^2^ = 63%, *P = 0.02*; Fig. S4).

### Contacts with pets

**Figure 3:**
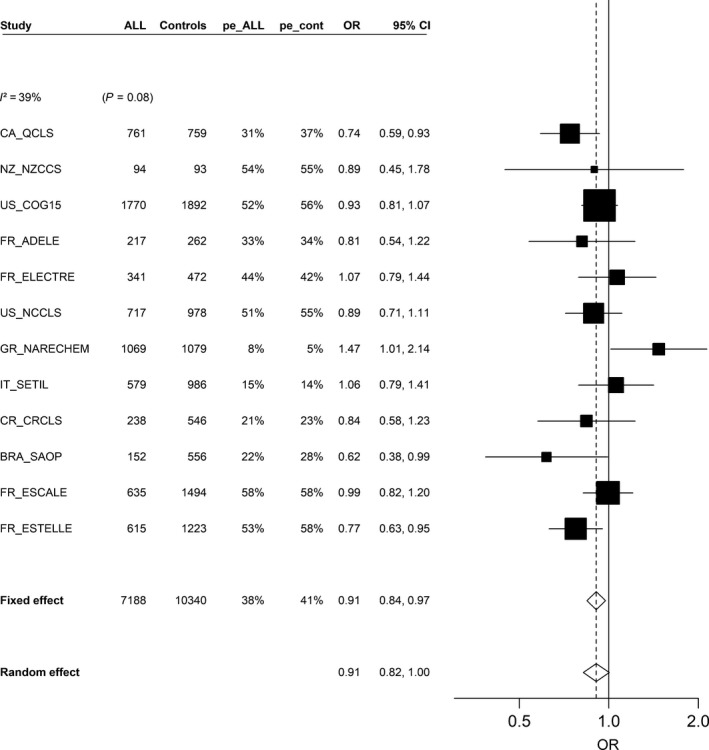
Association between acute lymphoblastic leukemia and contact with any pet in the first year of life (yes vs. no), (restricted to children aged ≥1 year), meta‐analysis of 12 studies (1980–2013), Childhood Leukemia International Consortium. ALL, acute lymphoblastic leukemia; BRA_SAOP, State of Sao Paulo Childhood Acute Lymphoblastic Leukemia Study (Brasil); CA_QCLS, Quebec Childhood Leukemia Study (Canada); CI, confidence interval; CR_CRLS, Costa Rican Childhood Leukemia Study (Costa Rica); FR_ADELE, Adele study (France); FR_ELECTRE, Electre Study (France); FR_ESCALE, Epidemiological Study on Childhood Cancer and Leukemia (France); FR_ESTELLE, Epidemiologic Study on Childhood Cancer, Leukemia and lymphoma (France); GR_NARECHEM, Nationwide Registration for Childhood Hematological Malignancies (Greece); IT_SETIL, Study on the Etiology of Childhood Lymphohematopoietic Malignancies (Italy); NZ_NZCCS, New Zealand Childhood Cancer Study (New Zealand); OR, odds ratio; Pe_ALL, prevalence of exposure among acute lymphoblastic leukemia cases; Pe_cont, prevalence of exposure among controls; US_COG15, Children's Oncology Group Study (US); US_NCCLS, Northern California Childhood Leukemia Study (US). Studies are ordered by increasing study period. Study‐specific odds ratios and 95% confidence interval were estimated by conditional (CA_QCLS, GR_NARECHEM, NZ_NZCCS, US_COG15, US_NCCLS) or unconditional (BRA_SAOP, CR_CRLS, FR_ADELE, FR_ELECTRE, FR_ESCALE, FR_ESTELLE, IT_SETIL) logistic models, adjusted for child's age at reference date, sex, maternal educational level (BRA_SAOP, FR_ELECTRE, GR_NARECHEM, IT_SETIL, NZ_NZCCS, US_COG15, US_NCCLS), ethnicity (FR_ADELE, NZ_NZCCS, US_COG15), region or center of recruitment (FR_ADELE), region or state of residence (FR_ELECTRE), “urban/rural” status of the place of residence (GR_NARECHEM, FR_ESTELLE), parental professional category (BRA_SAOP, CR_CRLS, FR_ELECTRE, FR_ESCALE, FR_ESTELLE, GR_NARECHEM), household income (US_COG15, US_NCCLS), maternal age at child's birth (BRA_SAOP, CA_QCLS, FR_ELECTRE, FR_ESCALE, FR_ESTELLE, US_NCCLS).

The ORs for contact with dogs or cats in the first year of life were 0.92 (95% CI: 0.86, 0.99) and 0.87 (95% CI: 0.80, 0.94), respectively (Table 4). The meta‐analysis estimates were very close to those of the pooled analysis, and between‐study heterogeneity was seen for overall contact with pets (*I* ^2^ = 39%, *P = 0.08*; Fig. 3), but not for contact with cats or dogs (Figs. S5 and S6).

**Table 4:** Association between acute lymphoblastic leukemia and contact with pets in the first year of life, pooled analysis of 12 studies (1980–2013), children aged 1 to 14 years old, Childhood Leukemia International Consortium

	Number of studies	ALL (n = 7468)		Controls (n = 10,821)		OR^a^	95%CI	P
		*n*	%	*n*	%			
Contact with dogs	12^b^							
No		5287	72	7410	69	1.00	Ref.	
Yes		2087	28	3307	31	0.92	0.86, 0.99	0.02
Missing		94		104				
Contact with cats	12^b^							
No		5953	81	8426	78	1.00	Ref.	
Yes		1428	19	2311	22	0.87	0.80, 0.94	<0.001
Missing		87		84				
Contact with any pets	12^b^							
No		4531	62	6306	59	1.00	Ref.	
Yes		2815	38	4388	41	0.90	0.84, 0.96	0.002
Only dogs		1383	19	2075	19	0.94	0.87, 1.03	
Only cats		723	10	1078	10	0.89	0.80, 0.98	
Dog and cats		700	10	1230	12	0.82	0.74, 0.92	
Missing		122		127				

ALL, acute lymphoblastic leukemia; BRA_SAOP, State of Sao Paulo Childhood Acute Lymphoblastic Leukemia Study (Brazil); CA_QCLS, Quebec Childhood Leukemia Study (Canada); CI, confidence interval; CR_CRCLS, Costa Rican Childhood Leukemia Study (Costa Rica); FR_ADELE, Adele Study (France); FR_ELECTRE, Electre Study (France); FR_ESCALE, Epidemiologic Study on Childhood Cancer and Leukemia (France); FR_ESTELLE, Epidemiologic Study on Childhood Cancer, Leukemia and Lymphoma (France); GR_NARECHEM, Nationwide Registration for Childhood Hematological Malignancies (Greece); IT_SETIL, Study on the Etiology of Childhood Lymphohematopoietic Malignancies (Italy); NZ_NZCCS, New Zealand Childhood Cancer Study (New Zealand); OR, odds ratio; US_COG15, Children's Oncology Group Study (United States); US_NCCLS, Northern California Childhood Leukemia Study (United States).

a Odds ratio and 95% confidence interval (95%CI) were estimated by unconditional logistic regression adjusted for child's age at reference date, child's sex, maternal age at child's birth, maternal educational level, and study of origin.

b 12 studies: BRA_SAOP, CA_QCLS, CR_CRCLS, FR_ADELE, FR_ELECTRE, FR_ESCALE, FR_ESTELLE, GR_NARECHEM, IT_SETIL, NZ_NZCCS, US_COG15, US_NCCLS.

### Subgroup analyses

Results were similar by types of ALL, B lineage or T lineage. For B lineage ALL, the estimates were also similar by strata of age (Table S2).

### Joint exposure to farm residence and animals

The results were not substantially modified when the exposures were two by two included in the same model (Table 5). Nevertheless, although based on half of the studies that had this information, contact with pets was no longer associated with ALL when adjusted for contact with livestock (OR = 0.99, 95% CI: 0.89, 1.10) or for contact with poultry (OR = 0.99, 95% CI: 0.89, 1.11). There was a significant interaction between farm residence and contact with any pets in the first year of life (*P*_int_ = 0.01; Table** 5). Joint exposure to early contact with pets and living on a farm in the first year of life was associated with a decreased risk (OR = 0.67, 95% CI: 0.47, 0.96; not tabulated). The association between early contact with livestock and ALL did not differ strongly according to farm residence at young age. By contrast, the association with livestock contact in the first year of life was specifically restricted to children who were reported to have had contact with pets in the first year of life (OR = 0.60, 95% CI: 0.44, 0.81; not tabulated), although the interaction was marginally statistically significant (*P*_int_ = 0.09; Table 5).

**Table 5:** Joint association between acute lymphoblastic leukemia and living on a farm, regular contact with pets and livestock and poultry in the first year of life, pooled analyses of nine studies (1980–2013), children aged 1 to 14 years old, Childhood Leukemia International Consortium

	Number of studies	Model without interaction			Model with interaction		
		OR^a^	95% CI	P	OR^a^	95% CI	P
Living on a farm and contact with any livestock in the first year of life	4^b^						
Living on a farm in the first year of life (yes vs. no)		1.10	0.73, 1.65	0.64	1.07	0.66, 1.73	0.79
Contact with any livestock in the first year (yes vs. no)		0.43	0.29, 0.65	<0.001	0.42	0.26, 0.68	<0.001
Interaction					1.11	0.45, 2.74	0.82
Living on a farm and contact with any pets in the first year of life	6^c^						
Living on a farm in the first year of life (yes vs. no)		0.97	0.73, 1.30	0.86	1.78	1.03, 3.06	0.04
Contact with any pets in the first year of life (yes vs. no)		0.86	0.78, 0.95	0.004	0.88	0.79, 0.97	0.01
Interaction					0.43	0.23, 0.82	0.01
Contact with any pets and any livestock in the first year of life	7^d^						
Contact with any pets in the first year of life (yes vs. no)		0.99	0.89, 1.10	0.89	1.01	0.90, 1.12	0.92
Contact with any livestock in the first year of life (yes vs. no)		0.66	0.51, 0.87	0.003	1.08	0.59, 1.99	0.80
Interaction					0.55	0.28, 1.09	0.09
Living on a farm and contact with poultry in the first year of life	4^b^						
Living on a farm in the first year of life (yes vs. no)		0.89	0.61, 1.32	0.58	1.04	0.66, 1.63	0.87
Contact with poultry in the first year (yes vs. no)		0.65	0.48, 0.88	0.006	0.70	0.50, 0.98	0.04
Interaction					0.60	0.25, 1.42	0.24
Contact with any livestock and poultry in the first year of life	6^e^						
Contact with any livestock in the first year (yes vs. no)		0.67	0.48, 0.93	0.02	0.74	0.48, 1.14	0.17
Contact with poultry in the first year (yes vs. no)		0.92	0.70, 1.20	0.53	0.96	0.71, 1.31	0.80
Interaction					0.81	0.42, 1.54	0.52
Contact with any pets and poultry in the first year of life	6^e^						
Contact with any pets in the first year of life (yes vs. no)		0.99	0.89, 1.11	0.90	1.01	0.90, 1.13	0.91
Contact with poultry in the first year of life (yes vs. no)		0.80	0.63, 1.02	0.07	1.20	0.68, 2.13	0.54
Interaction					0.61	0.33, 1.15	0.13

ALL, acute lymphoblastic leukemia; CA_QCLS, Quebec Childhood Leukemia Study (Canada); CI, confidence interval; CR_CRCLS, Costa Rican Childhood Leukemia Study (Costa Rica); FR_ADELE, Adele Study (France); FR_ELECTRE, Electre Study (France); FR_ESCALE, Epidemiologic Study on Childhood Cancer and Leukemia (France); FR_ESTELLE, Epidemiologic Study on Childhood Cancer, Leukemia and Lymphoma (France); GR_NARECHEM, Nationwide Registration for Childhood Hematological Malignancies (Greece); NZ_NZCCS, New Zealand Childhood Cancer Study (New Zealand); OR, odds ratio; US_NCCLS, Northern California Childhood Leukemia Study (United States).

a Odds ratio and 95% confidence interval were estimated by unconditional logistic regression adjusted for child's age at reference date, child's sex, maternal age at child's birth, maternal educational level, and study of origin.

b Four studies: FR_ADELE, FR_ESCALE, FR_ESTELLE, NZ_NZCCS.

c Six studies: CA_QCLS, FR_ADELE, FR_ESCALE, FR_ESTELLE, NZ_NZCCS, US_NCCLS.

d Seven studies: CR_CRCLS, FR_ADELE, FR_ELECTRE, FR_ESCALE, FR_ESTELLE, GR_NARECHEM, NZ_NZCCS.

e Six studies: FR_ADELE, FR_ELECTRE, FR_ESCALE, FR_ESTELLE, GR_NARECHEM, NZ_NZCCS.

### Sensitivity analysis (pooled analysis)

Results from one‐stage meta‐analyses were similar to those from the pooled analysis (Table S3). Excluding one study at a time from the pooled analyses had no substantial impact on the results except for the associations with contacts with animals in the first year of life, which were close to the null when we excluded the FR_ESCALE study for livestock (OR = 0.80, 95% CI: 0.58, 1.11) and for cattle (OR = 0.68, 95% CI: 0.44, 1.05).

Restricting the analyses to population‐based case–control studies and adjusting the models for parental SES, early daycare attendance, and breastfeeding in the first year of life did not modify the observed associations (data not shown).

The deterministic sensitivity analyses for home pesticide use (Table S4) and paternal smoking (Table S5) as uncontrolled confounders showed that adjustment for those variables would lead to similar results.

We evaluated the possibility that the inverse association with livestock was due to higher participation of exposed controls. For such a bias to generate an odds ratio of the observed magnitude (OR = 0.65), participation fractions would have had to be of 100% for exposed controls and 76% for nonexposed controls (given the average control participation fraction of 77% and exposure prevalence of 4% in this study).

## Discussion

The present study, based on the largest sample to date from 13 case–control studies, suggests that ALL risk is inversely associated with regular contact with livestock and pets in the first year of life. More specifically, we found inverse associations for regular contact with cattle, pigs, sheep, dogs, and cats. In contrast, our findings do not support an association between living on a farm before 1 year of age and ALL.

The existing literature about living on a farm and contact with farm animals (livestock, poultry) or pets is sparse 14, 15, 16, 17, 18, 19, 35, and all those with data on the first year of life are included in the present pooled analyses 16, 17, 18, 19.

Regarding contacts with pets and farm animals, the publications from the two French studies (FR_ESCALE 17, FR_ESTELLE 18) also focused on the contacts of the first year of life and showed inverse relationships with ALL. The US_COG15 study 16 found no association, and this result remained when we recalculated the odds ratio for contacts during the first year specifically.

By contrast with our findings, two earlier studies not included in the current analyses, conducted in the USA 14 and Greece 15 reported significant positive associations between childhood leukemia and regular contact with pets during childhood 15 or between ALL and cat ownership 14. Nevertheless, the periods of exposure were different from those used in the present study and the estimates of relative risks were based on small numbers of ALL cases. Finally, an Israeli case–control study, with a broad definition for pet exposure (presence of an animal in child's first home), found an inverse association with leukemias and lymphomas combined (OR = 0.62, 95% CI: 0.43, 0.90) 35.

In regard to living on a farm, data from all three previous reports have been included in the present analyses. Both the French studies 17, 18 which specifically examined the same time period as in our hypothesis, that is early childhood (in the first year of life), and the New Zealand one 19 had findings supportive of an inverse association between living on a farm and ALL.

To date, several studies are supportive of the hygiene hypothesis, with inverse associations reported between early contact with animal and asthma and other atopic conditions 36, 37, 38, respiratory tract illness 39. While the underlying mechanism leading to the protective effect of animal contact is not well understood, it has been speculated that early exposure to animals could help to mature the immune system 39. Several case–control studies have reported inverse associations between history of allergies, a marker of an abnormal immune response, and ALL 40. Although the biological mechanisms involved in that association are still unclear, it has been suggested that allergies and childhood acute leukemia might share common etiology 41.

Similarly, according to Greaves’ delayed infection hypothesis, early immune stimulations are suggested to be protective against ALL. Daycare attendance in the first year of life has been inversely associated to ALL in several studies, as reviewed in a meta‐analysis 7 and in a pooled study 10. Day care can be considered a proxy for exposures to common infectious agents, which contribute to maturation of the immune system. In the present study, contacts with animals are also viewed as opportunities for early stimulation of the immune system.

The major strength of these current analyses was the large sample size. While some of the studies had previously published their findings, we were able to include nine unpublished studies with 4655 cases. In addition, the access to the original data allowed us to harmonize the data to the same time window of interest, unlike in the original publications.

However, our investigations had potential weaknesses.

Case selection by survival could have occurred. Nevertheless, to our knowledge, the exposure of interest is not expected to impact survival. The procedure for control selection varied between studies, and in particular, two studies (GR_NARECHEM and FR_ADELE) were hospital‐based whereas the others were population‐based. In the sensitivity analyses, the results were similar when the two hospital‐based studies were excluded or when each of the other studies was excluded in turn, which suggests that biases inherent to hospital‐based studies or to one study in particular are not likely to explain the associations. Participation in controls was also high in the included studies. Moreover, the sensitivity analysis showed that a participation bias is unlikely to explain the results, as it would correspond to a differential participation of 100% of exposed controls and 76% on unexposed controls.

Recall bias regarding the exposure under study might have occurred, but it is not expected to be differential. Unlike parental tobacco or alcohol beverage consumption, contact with animals and residence in a farm are not exposures for which report may be accompanied by guilt and thus lead to systematic under‐reporting in case parents compared to control parents. Nondifferential errors may also have occurred, which could have biased estimates toward the null, particularly for farm residence, although the exposures of interest are not particularly difficult to remember. To reduce nondifferential errors, because of differences in questionnaires between studies, we used the most specific definition for exposure and kept separate the exposures which could not be aggregated. To deal with potential misclassification related to parent's recall, as exposure may have occurred up to 15 years before the interview, we performed stratified analyses on the child's age at diagnosis/interview, which generated similar results. Lack of specificity for the definition we have used for farm residence might also have generated nondifferential errors. Indeed, in the studies included in this analysis, no details regarding the type of farms were available, which could have added to the level of heterogeneity.

In regard to the pooled analysis, potential confounding by factors associated with child age and sex was taken into account when adjusting for these variables. Study of origin was also systematically adjusted for, in order to take into account unmeasured potential confounding factors related to differences between studies such as their designs or recruitment periods. Moreover, our models were systematically adjusted for maternal age at child's birth and maternal education, factors which were associated with both case–control status and the exposures under study. When we also adjusted for socioeconomic status, which is more prone to differences in definition between studies than maternal education, the results were similar. Proxies of early immune stimulation associated with ALL occurrence in a previous CLIC analysis were also taken into account as additional adjustment variables and led to similar results. Children in contact with pets may be exposed to insecticides applied to control fleas and ticks, which are suggested to be positively associated to ALL and not likely to explain the present results. Even if the data were not available for each study, potential confounding by pesticide exposure and paternal smoking was investigated and results from deterministic sensitivity analysis suggested that omitting those variables from the main analysis only led to weak bias in the estimates of the exposures under study.

The main disadvantage of the pooled analysis is that study‐specific adjustment variables and specific design such as case–control paired matching cannot be taken into account. However, the results were similar for the meta‐analyses in which adjustment was made for study‐specific stratification variables or confounding factors and conditional logistic models were performed to take into account case–control paired matching when necessary.

Finally, investigating the exposure related to animals in terms of *number of animals* in addition to the ever/never variables we have used would have helped to interpret our findings. Nevertheless, such data were only available for the FR_ADELE, FR_ELECTRE, and NZ_NZCCS studies, which precluded a robust analysis.

In conclusion, the findings of these large pooled and meta‐analyses bring additional evidence to the hypothesis that regular contact with animals in early childhood is inversely associated with childhood ALL occurrence. This is consistent with Greaves’ delayed infection hypothesis. Nevertheless, biological mechanisms involved in this association are still to be discovered.

## Conflict of Interest

The authors declare that they have no conflict of interest.

## Supporting information


**Figure S1.** Association between acute lymphoblastic leukemia and contact with cattle in the first year of life (yes vs. no), Restricted to children aged ≥1 year, meta‐analysis of 7 studies (1990–2013), Childhood Leukemia International Consortium.

**Figure S2.** Association between acute lymphoblastic leukemia and contact with pigs in the first year of life (yes vs. no), restricted to children aged ≥1 year, meta‐analysis of 7 studies (1990–2013), Childhood Leukemia International Consortium.

**Figure S3.** Association between acute lymphoblastic leukemia and contact with sheep in the first year of life (yes vs. no), restricted to children aged ≥1 year, meta‐analysis of 6 studies (1990–2013), Childhood Leukemia International Consortium.

**Figure S4.** Association between acute lymphoblastic leukemia and contact with poultry in the first year of life (yes vs. no), restricted to children aged ≥1 year, meta‐analysis of 6 studies (1990–2013), Childhood Leukemia International Consortium.

**Figure S5.** Association between Acute Lymphoblastic Leukemia and Contact with Dogs in the First Year of Life (yes vs. no), Restricted to Children Aged ≥1 year, Meta‐Analysis of 12 studies (1980–2013), Childhood Leukemia International Consortium.

**Figure S6.** Association between acute lymphoblastic leukemia and contact with cats in the first year of life (yes vs. no), restricted to children aged ≥1 year, meta‐analysis of 12 studies (1980–2013), Childhood Leukemia International Consortium.

**Table S1.** Description of exposures of interest among controls, children aged 1 to 14 years old from all studies combined (1980–2013), Childhood Leukemia International Consortium.
**Table S2.** Association between ALL and living in a farm, regular contact with animals in the first year of life, pooled stratified analyses of 11 studies (1980–2013) by ALL subtypes and by age, children aged 1 to14 years old, Childhood Leukemia International Consortium.
**Table S3.** Association between ALL and living in a farm, regular contact with animals in the first year of life, pooled and one‐stage meta‐analyses of 13 studies (1980–2013), children aged 1 to 14 years old, Childhood Leukemia International Consortium.
**Table S4.** Deterministic sensitivity analyses for home maternal pesticide use during pregancy as an Uncontrolled Confounder in the Investigations of the Association between Farm Residence, contact with livestock and pets in early childhood and ALL.
**Table S5.** Deterministic sensitivity analyses for paternal smoking as an Uncontrolled Confounder in the Investigations of the Association between farm residence, contact with livestock and pets in early childhood and ALL.

**Appendix S1.** Acknowledgements and Funding by study.

## References

[1] Kaatsch, P. , E. Steliarova‐Foucher , E. Crocetti , C. Magnani , C. Spix , and P. Zambon . 2006 Time trends of cancer incidence in European children (1978–1997): report from the Automated Childhood Cancer Information System project. Eur. J. Cancer 42:1961–1971. 16919764 10.1016/j.ejca.2006.05.014

[2] Baade, P. D. , D. R. Youlden , P. C. Valery , T. Hassall , L. Ward , A. C. Green , et al. 2010 Trends in incidence of childhood cancer in Australia, 1983–2006. Br. J. Cancer 102:620–626. 20051948 10.1038/sj.bjc.6605503 PMC2822940

[3] Lacour, B. , A. Guyot‐Goubin , S. Guissou , S. Bellec , E. Desandes , and J. Clavel . 2010 Incidence of childhood cancer in France: National Children Cancer Registries, 2000–2004. Eur. J. Cancer Prev. 19:173–181. 20361423 10.1097/cej.0b013e32833876c0

[4] Ward, E. , C. DeSantis , A. Robbins , B. Kohler , and A. Jemal . 2014 Childhood and adolescent cancer statistics, 2014. CA Cancer J. Clin. 64:83–103. 24488779 10.3322/caac.21219

[5] Whitehead, T. P. , C. Metayer , J. L. Wiemels , A. W. Singer , and M. D. Miller . 2016 Childhood leukemia and primary prevention. Curr. Probl. Pediatr. Adolesc. Health Care 46:317–352. 27968954 10.1016/j.cppeds.2016.08.004 PMC5161115

[6] Greaves, M. F. 1988 Speculations on the cause of childhood acute lymphoblastic leukemia. Leukemia 2:120–125. 3278171

[7] Urayama, K. Y. , P. A. Buffler , E. R. Gallagher , J. M. Ayoob , and X. Ma . 2010 A meta‐analysis of the association between day‐care attendance and childhood acute lymphoblastic leukaemia. Int. J. Epidemiol. 39:718–732. 20110276 10.1093/ije/dyp378 PMC2878455

[8] Kwan, M. L. , P. A. Buffler , B. Abrams , and V. A. Kiley . 2004 Breastfeeding and the risk of childhood leukemia: a meta‐analysis. Public Health Rep. 119:521–535. 15504444 10.1016/j.phr.2004.09.002 PMC1497668

[9] Amitay, E. L. , and L. Keinan‐Boker . 2015 Breastfeeding and childhood leukemia incidence: a meta‐analysis and systematic review. JAMA Pediatr. 169:e151025. 26030516 10.1001/jamapediatrics.2015.1025

[10] Rudant, J. , T. Lightfoot , K. Y. Urayama , E. Petridou , J. D. Dockerty , C. Magnani , et al. 2015 Childhood acute lymphoblastic leukemia and indicators of early immune stimulation: a childhood leukemia international consortium study. Am. J. Epidemiol. 181:549–562. 25731888 10.1093/aje/kwu298 PMC4850899

[11] Metayer, C. , E. Milne , J. Clavel , C. Infante‐Rivard , E. Petridou , M. Taylor , et al. 2013 The Childhood Leukemia International Consortium. Cancer Epidemiol. 37:336–347. 23403126 10.1016/j.canep.2012.12.011 PMC3652629

[12] Brooks, C. , N. Pearce , and J. Douwes . 2013 The hygiene hypothesis in allergy and asthma: an update. Curr. Opin. Allergy Clin. Immunol. 13:70–77. 23103806 10.1097/ACI.0b013e32835ad0d2

[13] Subbarao, P. , P. J. Mandhane , and M. R. Sears . 2009 Asthma: epidemiology, etiology and risk factors. CMAJ 181:E181–E190. 19752106 10.1503/cmaj.080612 PMC2764772

[14] Buckley, J. D. , C. M. Buckley , K. Ruccione , H. N. Sather , M. J. Waskerwitz , W. G. Woods , et al. 1994 Epidemiological characteristics of childhood acute lymphocytic leukemia. Analysis by immunophenotype. The Childrens Cancer Group. Leukemia 8:856–864. 8182942

[15] Petridou, E. , D. Trichopoulos , V. Kalapothaki , A. Pourtsidis , M. Kogevinas , M. Kalmanti , et al. 1997 The risk profile of childhood leukaemia in Greece: a nationwide case‐control study. Br. J. Cancer 76:1241–1247. 9365177 10.1038/bjc.1997.541 PMC2228112

[16] Swensen, A. R. , J. A. Ross , X. O. Shu , G. H. Reaman , M. Steinbuch , and L. L. Robison . 2001 Pet ownership and childhood acute leukemia (USA and Canada). Cancer Causes Control 12:301–303. 11456225 10.1023/a:1011276417369

[17] Rudant, J. , L. Orsi , F. Menegaux , A. Petit , A. Baruchel , Y. Bertrand , et al. 2010 Childhood acute leukemia, early common infections, and allergy: The ESCALE Study. Am. J. Epidemiol. 172:1015–1027. 20807738 10.1093/aje/kwq233

[18] Ajrouche, R. , J. Rudant , L. Orsi , A. Petit , A. Baruchel , A. Lambilliotte , et al. 2015 Childhood acute lymphoblastic leukaemia and indicators of early immune stimulation: the Estelle study (SFCE). Br. J. Cancer 112:1017–1026. 25675150 10.1038/bjc.2015.53 PMC4366894

[19] Dockerty, J. D. , D. C. Skegg , J. M. Elwood , G. P. Herbison , D. M. Becroft , and M. E. Lewis . 1999 Infections, vaccinations, and the risk of childhood leukaemia. Br. J. Cancer 80:1483–1489. 10424755 10.1038/sj.bjc.6690548 PMC2363060

[20] Milne, E. , J. A. Royle , N. H. de Klerk , E. Blair , H. Bailey , C. Cole , et al. 2009 Fetal growth and risk of childhood acute lymphoblastic leukemia: results from an Australian case‐control study. Am. J. Epidemiol. 170:221–228. 19478236 10.1093/aje/kwp117

[21] Wunsch‐Filho, V. , D. M. Pelissari , F. E. Barbieri , L. Sant'Anna , C. T. de Oliveira , J. F. de Mata , et al. 2011 Exposure to magnetic fields and childhood acute lymphocytic leukemia in Sao Paulo, Brazil. Cancer Epidemiol. 35:534–539. 21840286 10.1016/j.canep.2011.05.008

[22] Infante‐Rivard, C. , J. Siemiatycki , R. Lakhani , and L. Nadon . 2005 Maternal exposure to occupational solvents and childhood leukemia. Environ. Health Perspect. 113:787–792. 15929905 10.1289/ehp.7707 PMC1257608

[23] Monge, P. , C. Wesseling , J. Guardado , I. Lundberg , A. Ahlbom , K. P. Cantor , et al. 2007 Parental occupational exposure to pesticides and the risk of childhood leukemia in Costa Rica. Scand. J. Work Environ. Health 33:293–303. 17717622 10.5271/sjweh.1146

[24] Clavel, J. , S. Bellec , S. Rebouissou , F. Menegaux , J. Feunteun , C. Bonaiti‐Pellie , et al. 2005 Childhood leukaemia, polymorphisms of metabolism enzyme genes, and interactions with maternal tobacco, coffee and alcohol consumption during pregnancy. Eur. J. Cancer Prev. 14:531–540. 16284498 10.1097/00008469-200512000-00007

[25] Jourdan‐Da, S. N. , Y. Perel , F. Mechinaud , E. Plouvier , V. Gandemer , P. Lutz , et al. 2004 Infectious diseases in the first year of life, perinatal characteristics and childhood acute leukaemia. Br. J. Cancer 90:139–145. 14710221 10.1038/sj.bjc.6601384 PMC2395311

[26] Petridou, E. T. , A. Pourtsidis , N. Dessypris , K. Katsiardanis , M. Baka , M. Moschovi , et al. 2008 Childhood leukaemias and lymphomas in Greece (1996–2006): a nationwide registration study. Arch. Dis. Child. 93:1027–1032. 18676433 10.1136/adc.2007.133249

[27] Magnani, C. , S. Mattioli , L. Miligi , A. Ranucci , R. Rondelli , A. Salvan , et al. 2014 SETIL: Italian multicentric epidemiological case‐control study on risk factors for childhood leukaemia, non hodgkin lymphoma and neuroblastoma: study population and prevalence of risk factors in Italy. Ital. J. Pediatr. 40:103. 25539823 10.1186/s13052-014-0103-5 PMC4310183

[28] Bartley, K. , C. Metayer , S. Selvin , J. Ducore , and P. Buffler . 2010 Diagnostic X‐rays and risk of childhood leukaemia. Int. J. Epidemiol. 39:1628–1637. 20889538 10.1093/ije/dyq162 PMC2992630

[29] Cochran, W. G. 1954 The combination of estimates from different experiments. Biometrics 10:101–129.

[30] Higgins, J. P. , and S. G. Thompson . 2002 Quantifying heterogeneity in a meta‐analysis. Stat. Med. 21:1539–1558. 12111919 10.1002/sim.1186

[31] DerSimonian, R. , and N. Laird . 1986 Meta‐analysis in clinical trials. Control. Clin. Trials 7:177–188. 3802833 10.1016/0197-2456(86)90046-2

[32] Bailey, H. D. , C. Infante‐Rivard , C. Metayer , J. Clavel , T. Lightfoot , P. Kaatsch , et al. 2015 Home pesticide exposures and risk of childhood leukemia: findings from the childhood leukemia international consortium. Int. J. Cancer 137:2644–2663. 26061779 10.1002/ijc.29631 PMC4572913

[33] Liu, R. , L. Zhang , C. M. McHale , and S. K. Hammond . 2011 Paternal smoking and risk of childhood acute lymphoblastic leukemia: systematic review and meta‐analysis. J. Oncol. 2011:854584. 21765828 10.1155/2011/854584 PMC3132639

[34] Orsini, N. , R. Bellocco , M. Bottai , and A. Wolk . 2008 A tool for deterministic and probabilistic sensitivity analysis of epidemiological studies. Stata J. 8:29–48.

[35] Amitay, E. L. , R. G. Dubnov , and L. Keinan‐Boker . 2016 Breastfeeding, other early life exposures and childhood leukemia and lymphoma. Nutr. Cancer 68:968–977. 27352124 10.1080/01635581.2016.1190020

[36] Boneberger, A. , D. Haider , J. Baer , L. Kausel , R. von Kries , M. Kabesch , et al. 2011 Environmental risk factors in the first year of life and childhood asthma in the Central South of Chile. J. Asthma 48:464–469. 21548831 10.3109/02770903.2011.576740

[37] Fall, T. , C. Lundholm , A. K. Ortqvist , K. Fall , F. Fang , A. Hedhammar , et al. 2015 Early exposure to dogs and farm animals and the risk of childhood asthma. JAMA Pediatr. 169:e153219. 26523822 10.1001/jamapediatrics.2015.3219

[38] Bufford, J. D. , C. L. Reardon , Z. Li , K. A. Roberg , D. DaSilva , P. A. Eggleston , et al. 2008 Effects of dog ownership in early childhood on immune development and atopic diseases. Clin. Exp. Allergy 38:1635–1643. 18702654 10.1111/j.1365-2222.2008.03018.x

[39] Bergroth, E. , S. Remes , J. Pekkanen , T. Kauppila , G. Buchele , and L. Keski‐Nisula . 2012 Respiratory tract illnesses during the first year of life: effect of dog and cat contacts. Pediatrics 130:211–220. 22778307 10.1542/peds.2011-2825

[40] Linabery, A. M. , A. M. Jurek , S. Duval , and J. A. Ross . 2010 The association between atopy and childhood/adolescent leukemia: a meta‐analysis. Am. J. Epidemiol. 171:749–764. 20228139 10.1093/aje/kwq004 PMC2877483

[41] Chang, J. S. , J. L. Wiemels , and P. A. Buffler . 2009 Allergies and childhood leukemia. Blood Cells Mol. Dis. 42:99–104. 19049852 10.1016/j.bcmd.2008.10.003

