# Supplementary figures and images for: Living on a farm, contact with farm animals and pets, and childhood acute lymphoblastic leukemia: pooled and meta‐analyses from the Childhood Leukemia International Consortium
Source: Cancer Med. 2018 Apr 16;7(6):2665–81. doi: 10.1002/cam4.1466 (PMC6010788; doi:10.1002/cam4.1466)

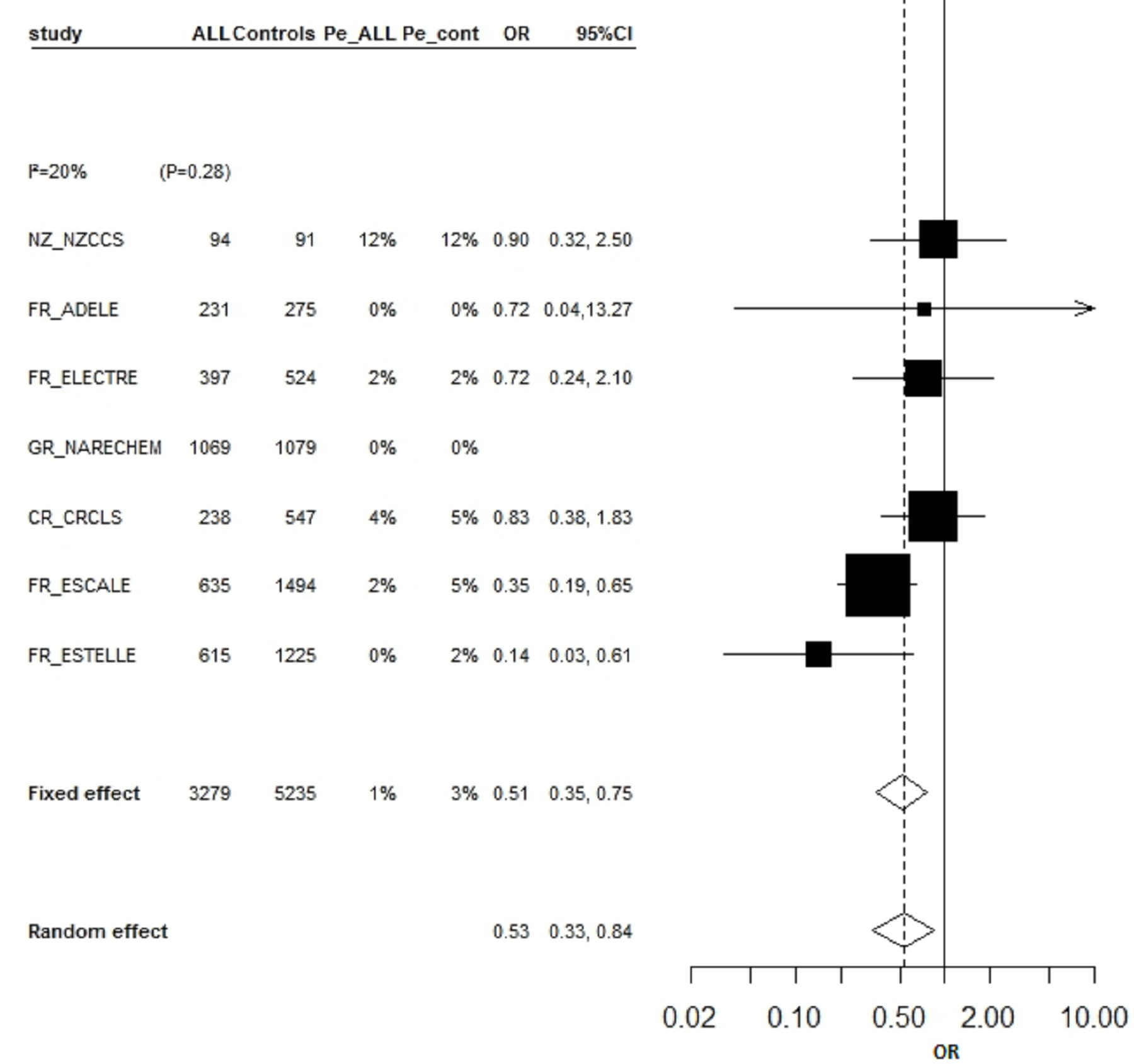

Supplement: Supplementary file 1 — Figure S1. Association between acute lymphoblastic leukemia and contact with cattle in the first year of life (yes vs. no), Restricted to children aged ≥1 year, meta‐analysis of 7 studies (1990–2013), Childhood Leukemia International Consortium. [file CAM4-7-2665-s001.tiff]

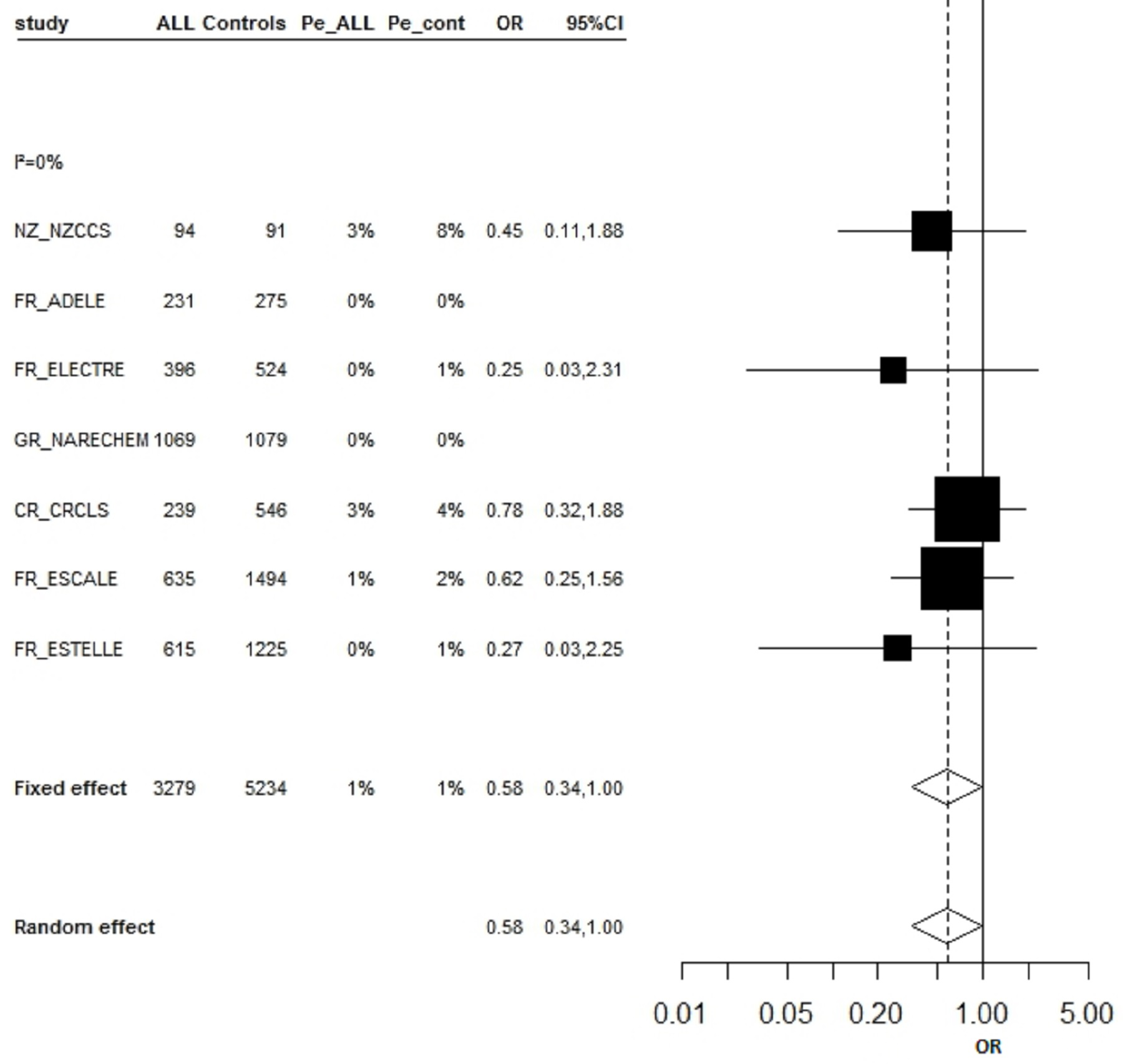

Supplement: Supplementary file 2 — Figure S2. Association between acute lymphoblastic leukemia and contact with pigs in the first year of life (yes vs. no), restricted to children aged ≥1 year, meta‐analysis of 7 studies (1990–2013), Childhood Leukemia International Consortium. [file CAM4-7-2665-s002.tiff]

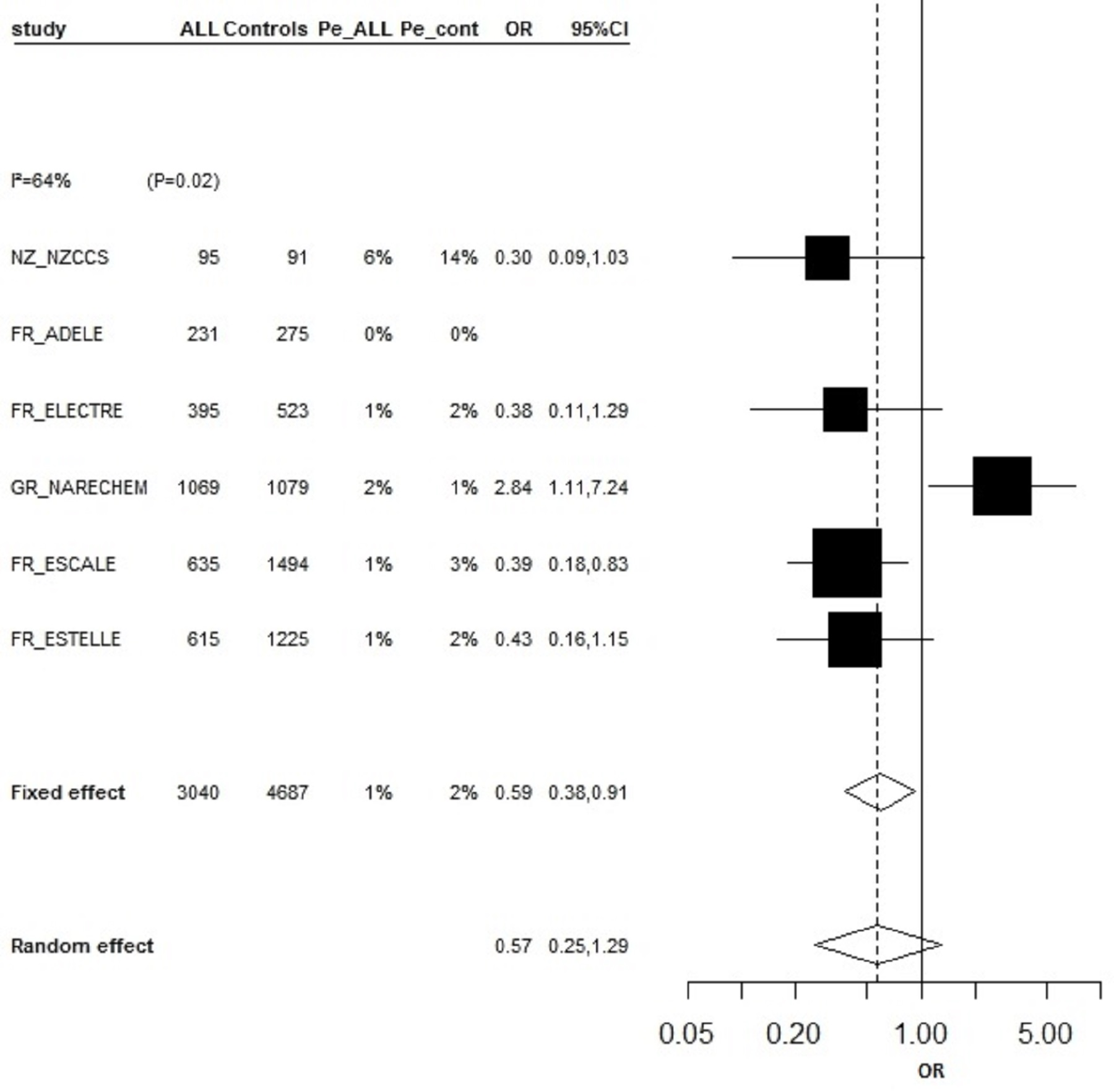

Supplement: Supplementary file 3 — Figure S3. Association between acute lymphoblastic leukemia and contact with sheep in the first year of life (yes vs. no), restricted to children aged ≥1 year, meta‐analysis of 6 studies (1990–2013), Childhood Leukemia International Consortium. [file CAM4-7-2665-s003.tiff]

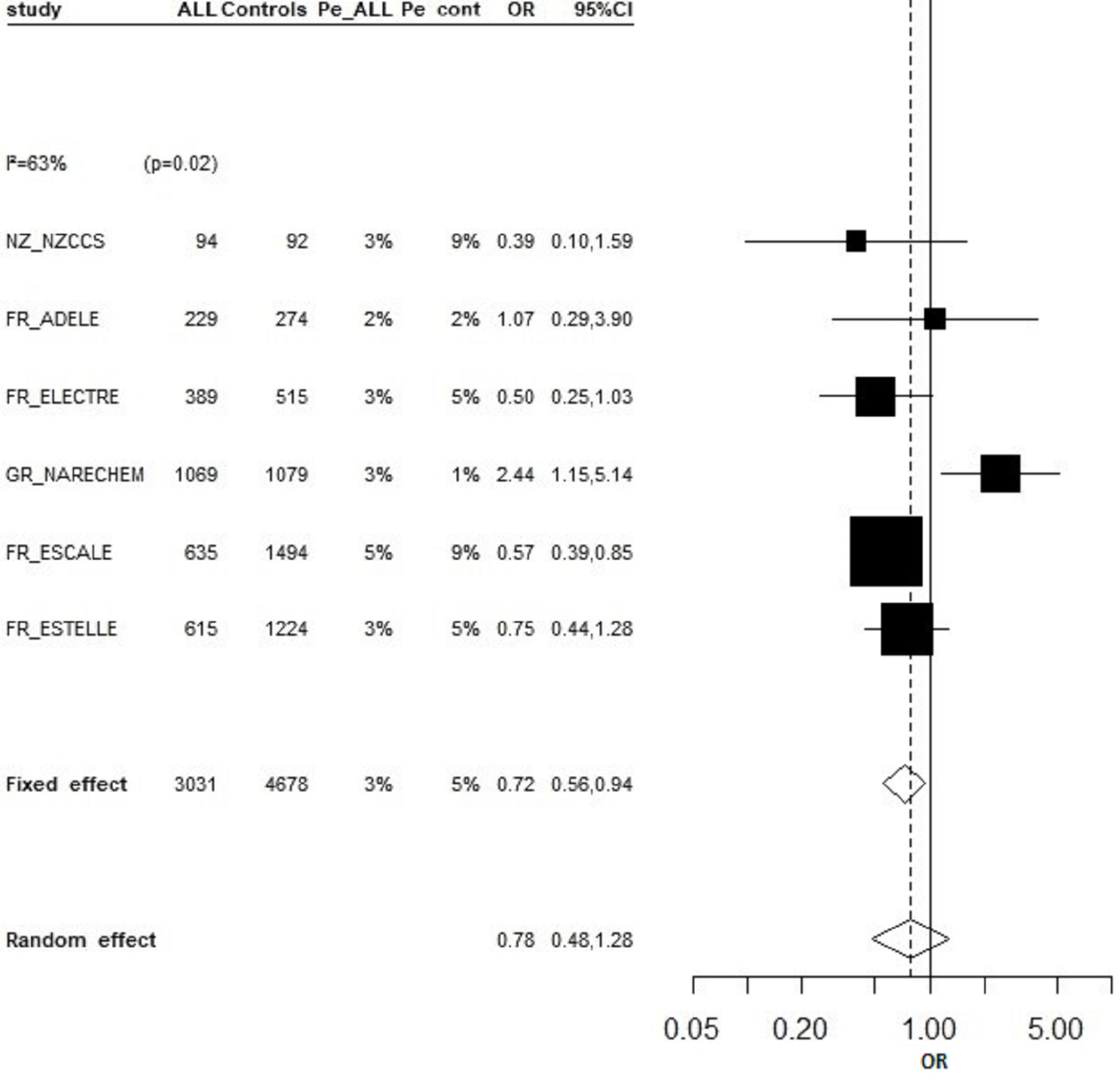

Supplement: Supplementary file 4 — Figure S4. Association between acute lymphoblastic leukemia and contact with poultry in the first year of life (yes vs. no), restricted to children aged ≥1 year, meta‐analysis of 6 studies (1990–2013), Childhood Leukemia International Consortium. [file CAM4-7-2665-s004.tiff]

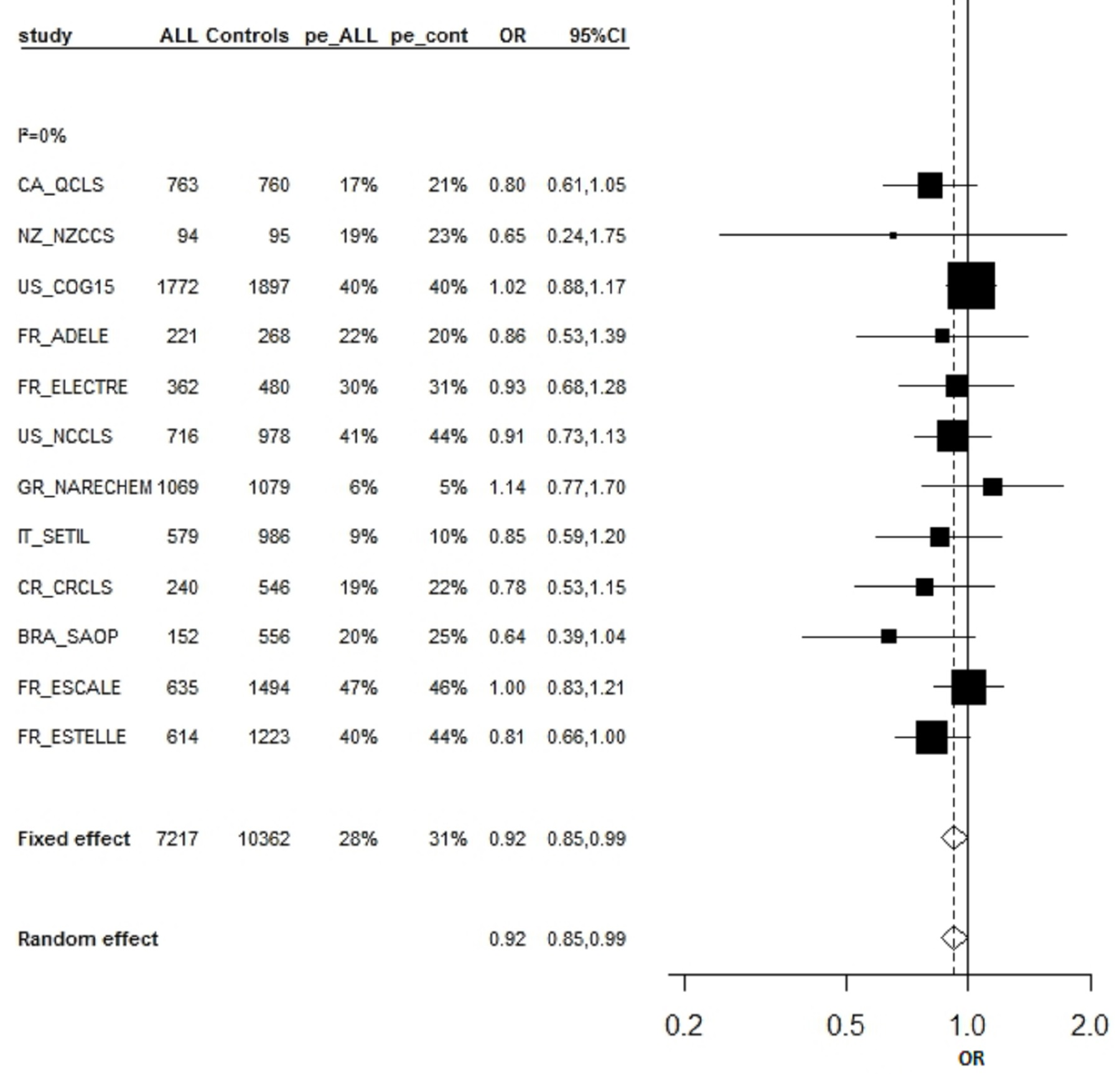

Supplement: Supplementary file 5 — Figure S5. Association between Acute Lymphoblastic Leukemia and Contact with Dogs in the First Year of Life (yes vs. no), Restricted to Children Aged ≥1 year, Meta‐Analysis of 12 studies (1980–2013), Childhood Leukemia International Consortium. [file CAM4-7-2665-s005.tiff]

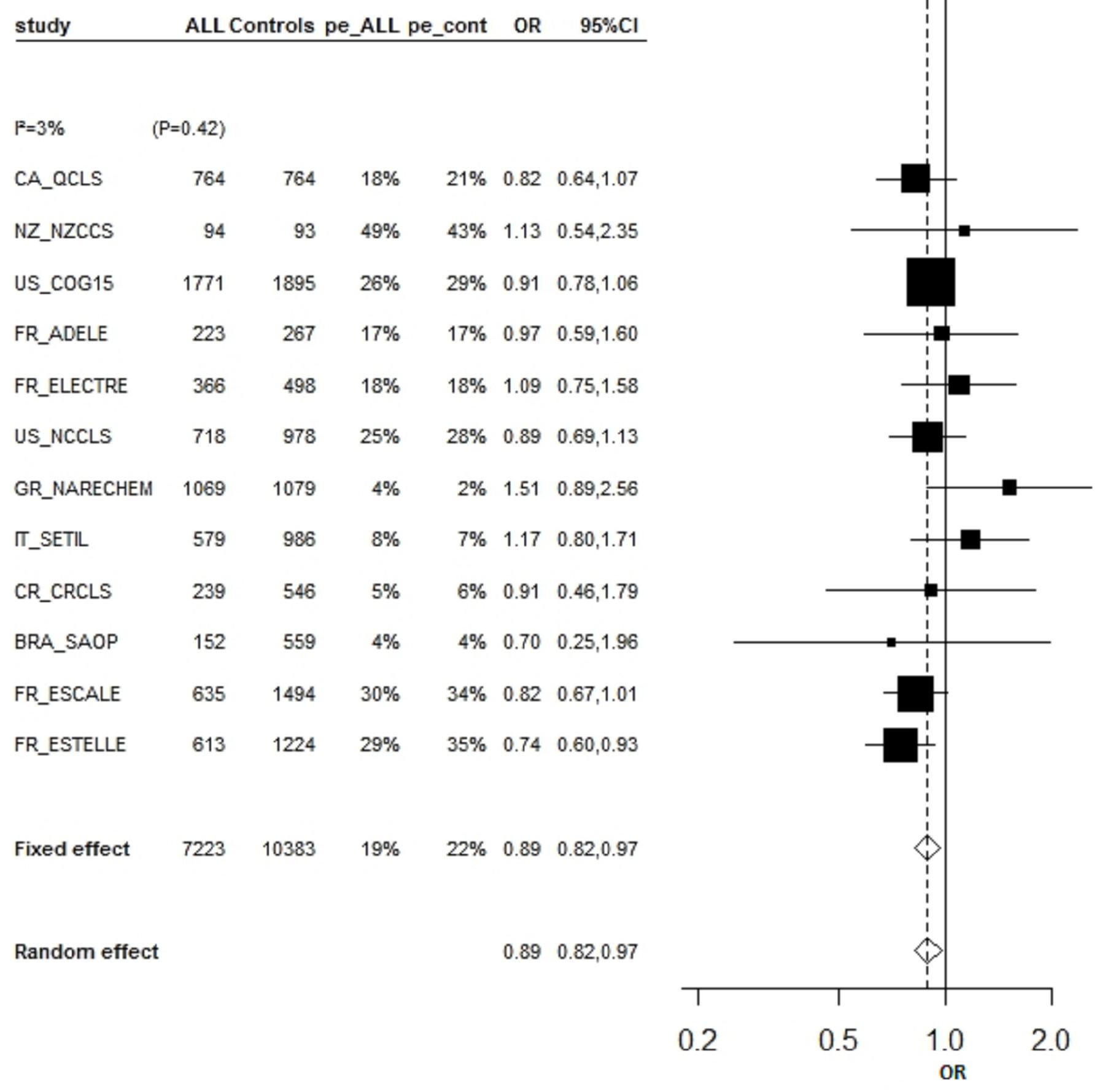

Supplement: Supplementary file 6 — Figure S6. Association between acute lymphoblastic leukemia and contact with cats in the first year of life (yes vs. no), restricted to children aged ≥1 year, meta‐analysis of 12 studies (1980–2013), Childhood Leukemia International Consortium. [file CAM4-7-2665-s006.tiff]
