# Supplementary material for: Living on a farm, contact with farm animals and pets, and childhood acute lymphoblastic leukemia: pooled and meta‐analyses from the Childhood Leukemia International Consortium
Source: Cancer Med. 2018 Apr 16;7(6):2665–81. doi: 10.1002/cam4.1466 (PMC6010788; doi:10.1002/cam4.1466)
Supplement: Supplementary file 7 — Table S1. Description of exposures of interest among controls, children aged 1 to 14 years old from all studies combined (1980–2013), Childhood Leukemia International Consortium. Table S2. Association between ALL and living in a farm, regular contact with animals in the first year of life, pooled stratified analyses of 11 studies (1980–2013) by ALL subtypes and by age, children aged 1 to14 years old, Childhood Leukemia International Consortium. Table S3. Association between ALL and living in a farm, regular contact with animals in the first year of life, pooled and one‐stage meta‐analyses of 13 studies (1980–2013), children aged 1 to 14 years old, Childhood Leukemia International Consortium. Table S4. Deterministic sensitivity analyses for home maternal pesticide use during pregancy as an Uncontrolled Confounder in the Investigations of the Association between Farm Residence, contact with livestock and pets in early childhood and ALL. Table S5. Deterministic sensitivity analyses for paternal smoking as an Uncontrolled Confounder in the Investigations of the Association between farm residence, contact with livestock and pets in early childhood and ALL. [file CAM4-7-2665-s007.doc]

**Web Table 1**. Description of Exposures of Interest among Controls, Children Aged 1 to 14 Years old from All Studies Combined (1980-2013), Childhood Leukemia International Consortium.

|  | **Maternal education** | | |  | **Socioeconomic status** | | |  | **Living on a farm (any definition)** | |  | **Contact with pets** | |
| --- | --- | --- | --- | --- | --- | --- | --- | --- | --- | --- | --- | --- | --- |
|  | Not secondary | Secondary | Tertiary |  | Low | Medium | High |  | No | Yes |  | No | Yes |
|  | 100% | 100% | 100% |  | 100% | 100% | 100% |  | 100% | 100% |  | 100% | 100% |
| **Living on a farm (any definitions)** |  |  |  |  |  |  |  |  |  |  |  |  |  |
| No | 96% | 97% | 98% |  | 95% | 97% | 98% |  |  |  |  |  |  |
| Yes | 4% | 3% | 2% |  | 5% | 3% | 2% |  |  |  |  |  |  |
| *P-value (Chi² test)* | *0.002* | | |  | *<0.0001* | | |  |  |  |  |  |  |
|  |  |  |  |  |  |  |  |  |  |  |  |  |  |
| **Contact with livestock in the first year of life** | |  |  |  |  |  |  |  |  |  |  |  |  |
| No | 95% | 96% | 97% |  | 96% | 95% | 97% |  | 98% | 53% |  | 99% | 92% |
| Yes | 5% | 4% | 3% |  | 4% | 5% | 3% |  | 2% | 47% |  | 1% | 8% |
| *P-value (Chi² test)* | *0.001* | | |  | *0.03* | | |  | *<0.0001* | |  | *<0.0001* | |
|  |  |  |  |  |  |  |  |  |  |  |  |  |  |
| **Contact with poultry in the first year of life** |  |  |  |  |  |  |  |  |  |  |  |  |  |
| No | 93% | 96% | 97% |  | 94% | 95% | 96% |  | 96% | 58% |  | 99% | 89% |
| Yes | 7% | 4% | 3% |  | 6% | 5% | 4% |  | 4% | 42% |  | 1% | 11% |
| *P-value (Chi² test)* | *<0.0001* | | |  | *0.04* | | |  | *<0.0001* | |  | *<0.0001* | |
| **Contact with pets in the first year of life** |  |  |  |  |  |  |  |  |  |  |  |  |  |
| No | 61% | 61% | 55% |  | 60% | 62% | 54% |  | 57% | 23% |  |  |  |
| Yes | 39% | 39% | 45% |  | 40% | 38% | 46% |  | 43% | 77% |  |  |  |
| *P-value (Chi² test)* | *<0.0001* | | |  | *<0.0001* | | |  | *<0.0001* | |  |  |  |

**Web Table 2.** Association between ALL and Living in a Farm, Regular Contact with Animals in the First Year of Life, Pooled Stratified Analyses of 11 studies (1980-2013) by ALL Subtypes and by Age, Children aged 1 to14 Years old, Childhood Leukemia International Consortium.

|  |  | **ALL subtypes 1-14 years** | | | | | |  | **B lineage ALL** | | | | | | |
| --- | --- | --- | --- | --- | --- | --- | --- | --- | --- | --- | --- | --- | --- | --- | --- |
|  |  | **B lineage ALL** | |  | **T lineage ALL** | | |  |  | **1-5 years** | |  | **6-14 years** | | |
|  | **n** | **OR^a^** | **95%CI** |  | **n** | **OR^a^** | **95%CI** |  | **n** | **OR^b^** | **95%CI** |  | **n** | **OR^b^** | **95%CI** |
| **Living on a farm (any definitions)^c^** |  |  |  |  |  |  |  |  |  |  |  |  |  |  |  |
| No | 3,439 | 1.00 | ref. |  | 426 | 1.00 | ref. |  | 2,282 | 1.00 | ref. |  | 1,157 | 1.00 | ref. |
| Yes | 114 | 1.06 | 0.83, 1.35 |  | 18 | 1.24 | 0.75, 2.05 |  | 65 | 1.03 | 0.75, 1.43 |  | 49 | 1.10 | 0.77, 1.58 |
| **Living on a farm in the first year of life^d^** |  |  |  |  |  |  |  |  |  |  |  |  |  |  |  |
| No | 2,181 | 1.00 | ref. |  | 285 | 1.00 | ref. |  | 1,469 | 1.00 | ref. |  | 712 | 1.00 | ref. |
| Yes | 70 | 0.96 | 0.71, 1.30 |  | 8 | 0.73 | 0.35, 1.54 |  | 39 | 0.90 | 0.60, 1.35 |  | 31 | 1.01 | 0.65, 1.58 |
|  |  |  |  |  |  |  |  |  |  |  |  |  |  |  |  |
| **Contact with livestock in the first year of life^e^** |  |  |  |  |  |  |  |  |  |  |  |  |  |  |  |
| No | 2,584 | 1.00 | ref. |  | 355 | 1.00 | ref. |  | 1,673 | 1.00 | ref. |  | 911 | 1.00 | ref. |
| Yes | 58 | 0.63 | 0.46, 0.85 |  | 11 | 0.87 | 0.46, 1.64 |  | 38 | 0.69 | 0.47, 1.03 |  | 20 | 0.57 | 0.34, 0.93 |
| *Contact with cattle^e^* |  |  |  |  |  |  |  |  |  |  |  |  |  |  |  |
| No | 2,613 | 1.00 | ref. |  | 364 | 1.00 | ref. |  | 1,689 | 1.00 | ref. |  | 924 | 1.00 | ref. |
| Yes | 33 | 0.54 | 0.37, 0.81 |  | 2 | 0.24 | 0.06, 0.96 |  | 23 | 0.61 | 0.37, 0.99 |  | 10 | 0.47 | 0.24, 0.93 |
| *Contact with pigs^e^* |  |  |  |  |  |  |  |  |  |  |  |  |  |  |  |
| No | 2,635 | 1.00 | ref. |  | 364 | 1.00 | ref. |  | 1,705 | 1.00 | ref. |  | 930 | 1.00 | ref. |
| Yes | 10 | 0.50 | 0.25, 1.01 |  | 2 | 0.76 | 0.18, 3.22 |  | 8 | 0.77 | 0.33, 1.82 |  | 2 | 0.24 | 0.05, 1.01 |
| *Contact with sheep^e^* |  |  |  |  |  |  |  |  |  |  |  |  |  |  |  |
| No | 2,610 | 1.00 | ref. |  | 357 | 1.00 | ref. |  | 1,690 | 1.00 | ref. |  | 920 | 1.00 | ref. |
| Yes | 35 | 0.62 | 0.41, 0.92 |  | 9 | 1.24 | 0.61, 2.52 |  | 23 | 0.70 | 0.42, 1.16 |  | 12 | 0.55 | 0.29, 1.05 |
| **Contact with poultry in the first year of life^e^** |  |  |  |  |  |  |  |  |  |  |  |  |  |  |  |
| No | 2,552 | 1.00 | ref. |  | 346 | 1.00 | ref. |  | 1,661 | 1.00 | ref. |  | 891 | 1.00 | ref. |
| Yes | 87 | 0.76 | 0.58, 0.98 |  | 17 | 1.00 | 0.60, 1.69 |  | 48 | 0.70 | 0.50, 1.00 |  | 39 | 0.86 | 0.59, 1.25 |
| **Contact with dogs^f^** |  |  |  |  |  |  |  |  |  |  |  |  |  |  |  |
| No | 3,990 | 1.00 | ref. |  | 555 | 1.00 | ref. |  | 2,702 | 1.00 | ref. |  | 1,288 | 1.00 | ref. |
| Yes | 1,564 | 0.93 | 0.86, 1.01 |  | 215 | 0.93 | 0.78, 1.11 |  | 1,067 | 0.94 | 0.85, 1.03 |  | 497 | 0.91 | 0.79, 1.04 |
| **Contact with cats^f^** |  |  |  |  |  |  |  |  |  |  |  |  |  |  |  |
| No | 4,452 | 1.00 | ref. |  | 635 | 1.00 | ref. |  | 3,005 | 1.00 | ref. |  | 1,447 | 1.00 | ref. |
| Yes | 1,108 | 0.88 | 0.81, 0.96 |  | 139 | 0.80 | 0.66, 0.98 |  | 772 | 0.90 | 0.81, 1.00 |  | 336 | 0.84 | 0.73, 0.98 |
| **Contact with pets in the first year of life** ^f^ |  |  |  |  |  |  |  |  |  |  |  |  |  |  |  |
| No | 3,412 | 1.00 | ref. |  | 483 | 1.00 | ref. |  | 2,296 | 1.00 | ref. |  | 1,116 | 1.00 | ref. |
| Yes | 2,120 | 0.91 | 0.84, 0.98 |  | 286 | 0.89 | 0.76, 1.05 |  | 1,462 | 0.90 | 0.82, 0.99 |  | 658 | 0.91 | 0.80, 1.03 |
| Only dog | 1009 | 0.95 | 0.86, 1.04 |  | 146 | 0.99 | 0.81, 1.22 |  | 689 | 0.92 | 0.82, 1.03 |  | 320 | 0.98 | 0.84, 1.15 |
| Only cat | 552 | 0.88 | 0.78, 0.98 |  | 70 | 0.85 | 0.65, 1.11 |  | 392 | 0.86 | 0.75, 0.99 |  | 160 | 0.92 | 0.75, 1.14 |
| Dog and cat | 552 | 0.84 | 0.75, 0.95 |  | 68 | 0.75 | 0.57, 0.98 |  | 377 | 0.89 | 0.77, 1.03 |  | 175 | 0.78 | 0.64, 0.94 |

Abbreviations: ALL, acute lymphoblastic leukemia; AUS_ALL, Australian Study of Causes of Acute Lymphoblastic Leukemia in Children; CA_QCLS, Quebec childhood leukemia study (Canada); CI, confidence interval; FR_ADELE, Adele study (France); FR_ELECTRE, Electre study (France); FR_ESCALE, Epidemiological study on childhood cancer and leukemia (France); FR_ESTELLE, Epidemiologic Study on Childhood Cancer, Leukemia and lymphoma (France); GR_NARECHEM, Nationwide registration for childhood haematological malignancies (Greece); IT_SETIL, Study on the etiology of childhood lymphohematopoietic malignancies (Italy); NZ_NZCCS, New Zealand childhood cancer study (New Zealand); OR, odds ratio; US_COG15, Children’s oncology group study (US); US_NCCLS, Northern California childhood leukemia study (US).

^a^Odds ratio and 95% confidence interval (95%CI) were estimated by polytomous logistic regression adjusted for child’s age at reference, child’s sex, maternal age at child’s birth, maternal educational level and study of origin.

^b^, Odds-Ratio (OR) and 95% Confidence Interval (95%CI) were estimated by unconditional logistic regression adjusted for child’s age at reference date, child’s sex, maternal age at child’s birth, maternal educational level, and study of origin.

^c^ 8 studies: AUS_ALL, CA_QCLS, FR_ADELE, FR_ESCALE, FR_ESTELLE, GR_NARECHEM, NZ_NZCCS, US_NCCLS.

^d^ 6 studies: CA_QCLS, FR_ADELE, FR_ESCALE, FR_ESTELLE, NZ_NZCCS, US_NCCLS.

^e^ 6 studies: FR_ADELE, FR_ELECTRE, FR_ESCALE, FR_ESTELLE, GR_NARECHEM, NZ_NZCCS.

^f^ 10 studies: CA_QCLS, FR_ADELE, FR_ELECTRE, FR_ESCALE, FR_ESTELLE, GR_NARECHEM, IT_SETIL, NZ_NZCCS, US_COG15, US_NCCLS.

**Web Table 3.** Association between ALL and Living in a Farm, Regular Contact with Animals in the First Year of Life, Pooled and one-stage Meta-Analyses of 13 studies (1980-2013), Children aged 1 to14 Years old, Childhood Leukemia International Consortium.

|  | **Number of studies** | **ALL** | **Controls** |  | **pooled analysis** | | |  | **One-stage meta-analysis with random effects** | | |
| --- | --- | --- | --- | --- | --- | --- | --- | --- | --- | --- | --- |
|  |  | **Prevalence of exposure (%)** | **Prevalence of exposure (%)** |  | **OR^a^** | **95%CI** | ***p*** |  | **OR^b^** | **95%CI** | ***p*** |
| Living on a farm (any definitions) | 8^c^ | 3 | 3 |  | 1.09 | 0.86, 1.36 | *0.48* |  | 1.08 | 0.85, 1.38 | *0.52* |
| Living on a farm in the first year of life | 6^d^ | 3 | 3 |  | 0.93 | 0.70, 1.24 | *0.64* |  | 0.92 | 0.65, 1.29 | *0.62* |
| Contact with livestock in the first year of life | 7^e^ | 2 | 4 |  | 0.65 | 0.50, 0.85 | *0.002* |  | 0.68 | 0.44, 1.05 | *0.08* |
| *Contact with cattle* | 7^e^ | 1 | 3 |  | 0.54 | 0.39, 0.77 | *<0.001* |  | 0.55 | 0.38, 0.81 | *0.04* |
| *Contact with pigs* | 7^e^ | 1 | 1 |  | 0.58 | 0.35, 0.98 | *0.04* |  | 0.58 | 0.34, 0.98 | *0.04* |
| *Contact with sheep* | 6^f^ | 1 | 2 |  | 0.68 | 0.47, 0.98 | *0.04* |  | 0.65 | 0.39, 1.07 | *0.09* |
| Contact with poultry in the first year of life | 6^f^ | 3 | 5 |  | 0.78 | 0.62, 1.00 | *0.05* |  | 0.81 | 0.54, 1.21 | *0.30* |
| Contact with any pets in the first year of life | 12^g^ | 38 | 41 |  | 0.90 | 0.84, 0.96 | *0.002* |  | 0.91 | 0.83, 1.00 | *0.05* |
| *Contact with dogs* | 12^g^ | 28 | 31 |  | 0.92 | 0.86, 0.99 | *0.02* |  | 0.92 | 0.85, 0.99 | *0.02* |
| *Contact with cats* | 12^g^ | 19 | 22 |  | 0.87 | 0.80, 0.94 | *<0.001* |  | 0.87 | 0.81, 0.94 | *<0.001* |

Abbreviations: ALL, acute lymphoblastic leukemia; AUS_ALL, Australian Study of Causes of Acute Lymphoblastic Leukemia in Children; BRA_SAOP, State of Sao Paulo Childhood acute lymphoblastic leukemia study (Brazil); CA_QCLS, Quebec Childhood Leukemia Study (Canada); CR_CRCLS, Costa Rican Childhood Leukemia Study (Costa Rica); FR_ADELE, Adele Study (France); FR_ELECTRE, Electre Study (France); FR_ESCALE, Epidemiologic Study on Childhood Cancer and Leukemia (France); FR_ESTELLE, Epidemiologic Study on Childhood Cancer, Leukemia and lymphoma (France); GR_NARECHEM, Nationwide Registration for Childhood Hematological Malignancies (Greece); IT_SETIL, Study on the Etiology of Childhood Lymphohematopoietic Malignancies (Italy); NZ_NZCCS, New Zealand Childhood Cancer Study; US_COG15, Children’s Oncology Group Study (United States); US_NCCLS, Northern California Childhood Leukemia Study (United States).

^a^, Odds ratio and 95% confidence interval were estimated by unconditional logistic regression adjusted for child’s age at reference date, child’s sex, maternal age at child’s birth, maternal educational level, and study of origin.

^b^, Odds ratio and 95% confidence interval were estimated by unconditional logistic regression adjusted for child’s age at reference date, child’s sex, maternal age at child’s birth and maternal educational level, between-study variability was modeled by including a random intercept and a random slope for the exposure of interest.

^c^ 8 studies: AUS_ALL, CA_QCLS, FR_ADELE, FR_ESCALE, FR_ESTELLE, GR_NARECHEM, NZ_NZCCS, US_NCCLS.

^d^ 6 studies: CA_QCLS, FR_ADELE, FR_ESCALE, FR_ESTELLE, NZ_NZCCS, US_NCCLS.

^e^ 7 studies: CR_CRCLS, FR_ADELE, FR_ELECTRE, FR_ESCALE, FR_ESTELLE, GR_NARECHEM, NZ_NZCCS.

^f^ 6 studies: FR_ADELE, FR_ELECTRE, FR_ESCALE, FR_ESTELLE, GR_NARECHEM, NZ_NZCCS.

^g^ 12 studies: BRA_SAOP, CA_QCLS, CR_CRCLS, FR_ADELE, FR_ELECTRE, FR_ESCALE, FR_ESTELLE, GR_NARECHEM, IT_SETIL, NZ_NZCCS, US_COG15, US_NCCLS.

**Web Table 4.** Deterministic Sensitivity Analyses for Home Maternal Pesticide Use during Pregancy as an Uncontrolled Confounder in the Investigations of the Association between Farm Residence, Contact with Livestock and Pets in Early Childhood and ALL.

|  |  | Estimate of Relative risk between maternal home pesticide use during pregnancy and childhood ALL^2^ |  |  |  |
| --- | --- | --- | --- | --- | --- |
| Estimates of prevalence of maternal home pesticide use during pregnancy^1^ | |  | OR 95% CI | | % Bias |
| Living in a farm in early childhood | |  |  |  |  |
| Exposed | Non-exposed |  |  |  |  |
|  |  |  | Adjusted^3^ | 1.09 0.86, 1.36 |  |
|  |  |  | Crude | 1.10 0.88, 1.38 |  |
|  |  |  | External adjusted^4,5^ | |  |
| 0.45 | 0.40 | 1.35 |  | 1.08 0.86, 1.35 | 2 |
| 0.50 | 0.35 | 1.35 |  | 1.05 0.84, 1.31 | 5 |
| 0.55 | 0.30 | 1.35 |  | 1.02 0.82, 1.27 | 8 |
|  |  |  |  |  |  |
| 0.45 | 0.40 | 1.40 |  | 1.08 0.86, 1.35 | 2 |
| 0.50 | 0.35 | 1.40 |  | 1.05 0.84, 1.31 | 5 |
| 0.55 | 0.30 | 1.40 |  | 1.01 0.81, 1.26 | 9 |
|  |  |  |  |  |  |
| 0.45 | 0.40 | 1.45 |  | 1.08 0.86, 1.35 | 2 |
| 0.50 | 0.35 | 1.45 |  | 1.04 0.83, 1.30 | 6 |
| 0.55 | 0.30 | 1.45 |  | 1.00 0.80, 1.25 | 10 |
|  |  |  |  |  |  |
| Regular contact with livestock in early childhood | | |  |  |  |
|  |  |  | Adjusted^3^ | 0.65 0.50, 0.85 |  |
|  |  |  | Crude | 0.58 0.45, 0.76 |  |
|  |  |  | External adjusted^4,5^ | |  |
| 0.55 | 0.45 | 1.35 |  | 0.57 0.44, 0.74 | 3 |
| 0.60 | 0.40 | 1.35 |  | 0.55 0.42, 0.71 | 6 |
| 0.65 | 0.35 | 1.35 |  | 0.53 0.41, 0.69 | 9 |
|  |  |  |  |  |  |
| 0.55 | 0.45 | 1.40 |  | 0.56 0.43, 0.73 | 3 |
| 0.60 | 0.40 | 1.40 |  | 0.54 0.42, 0.70 | 7 |
| 0.65 | 0.35 | 1.40 |  | 0.53 0.41, 0.69 | 11 |
|  |  |  |  |  |  |
| 0.55 | 0.45 | 1.45 |  | 0.56 0.43, 0.73 | 4 |
| 0.60 | 0.40 | 1.45 |  | 0.54 0.42, 0.70 | 8 |
| 0.65 | 0.35 | 1.45 |  | 0.52 0.40, 0.68 | 12 |
|  |  |  |  |  |  |
| Regular contact with any pets in early childhood | | |  |  |  |
|  |  |  |  |  |  |
|  |  |  | Adjusted^3^ | 0.90 0.84, 0.96 |  |
|  |  |  | Crude | 0.89 0.84, 0.95 |  |
|  |  |  | External adjusted^4,5^ | |  |
| 0.45 | 0.40 | 1.35 |  | 0.88 0.83, 0.94 | 2 |
| 0.50 | 0.35 | 1.35 |  | 0.85 0.80, 0.90 | 5 |
| 0.55 | 0.30 | 1.35 |  | 0.83 0.78, 0.88 | 8 |
|  |  |  |  |  |  |
| 0.45 | 0.40 | 1.40 |  | 0.88 0.83, 0.94 | 2 |
| 0.50 | 0.35 | 1.40 |  | 0.85 0.80, 0.90 | 5 |
| 0.55 | 0.30 | 1.40 |  | 0.82 0.77, 0.87 | 9 |
|  |  |  |  |  |  |
| 0.45 | 0.40 | 1.45 |  | 0.88 0.83, 0.94 | 2 |
| 0.50 | 0.35 | 1.45 |  | 0.84 0.79, 0.89 | 6 |
| 0.55 | 0.30 | 1.45 |  | 0.81 0.76, 0.86 | 10 |

^1^ Based on maternal pesticide use in a sample of CLIC studies

^2^ Based on published estimates from CLIC (Bailey HD, et al. Int J Cancer 2015;137(11):2644-63)

^3^ Adjusted for child’s age at reference date, child’s sex, maternal age at child’s birth, maternal educational level and study of origin

^4^ OR calculated using the Episensi procedure in Stata command (Orsini N et al 2008)

^5^ 95%CI calculated using the following formula: exp(ln(external adjusted OR) +/- 1.96* stand error(ln (external adjusted OR))

**Web Table 5.** Deterministic Sensitivity Analyses for Paternal Smoking as an Uncontrolled Confounder in the Investigations of the Association between Farm Residence, Contact with Livestock and Pets in Early Childhood and ALL.

|  |  | Estimate of Relative risk between paternal smoking and childhood ALL^2^ |  |  |  |
| --- | --- | --- | --- | --- | --- |
| Estimates of prevalence of paternal smoking^1^ | |  | OR 95% CI |  | % Bias |
| Living in a farm in early childhood | |  |  |  |  |
| Exposed | Non-exposed |  |  |  |  |
|  |  |  | Adjusted^3^ | 1.09 0.86, 1.36 | |
|  |  |  | Crude | 1.10 0.88, 1.38 | |
|  |  |  | External adjusted^4,5^ | |  |
| 0.30 | 0.55 | 1.10 |  | 1.13 0.90, 1.41 | -2 |
| 0.35 | 0.50 | 1.10 |  | 1.12 0.90, 1.40 | -1 |
| 0.40 | 0.45 | 1.10 |  | 1.11 0.89, 1.39 | 0 |
|  |  |  |  |  |  |
| 0.30 | 0.55 | 1.15 |  | 1.14 0.91, 1.42 | -3 |
| 0.35 | 0.50 | 1.15 |  | 1.12 0.90, 1.40 | -2 |
| 0.40 | 0.45 | 1.15 |  | 1.11 0.89, 1.39 | -1 |
|  |  |  |  |  |  |
| 0.30 | 0.55 | 1.20 |  | 1.15 0.92, 1.44 | -5 |
| 0.35 | 0.50 | 1.20 |  | 1.13 0.90, 1.41 | -3 |
| 0.40 | 0.45 | 1.20 |  | 1.11 0.89, 1.39 | -1 |
|  |  |  |  |  |  |
| Regular contact with livestock in early childhood | | |  |  |  |
|  |  |  | Adjusted^3^ | 0.65 0.50, 0.85 | |
|  |  |  | Crude | 0.58 0.45, 0.76 | |
|  |  |  | External adjusted^4,5^ | |  |
| 0.40 | 0.55 | 1.10 |  | 0.59 0.45, 0.77 | -1 |
| 0.45 | 0.50 | 1.10 |  | 0.59 0.45, 0.77 | -1 |
| 0.50 | 0.45 | 1.10 |  | 0.58 0.45, 0.75 | 0 |
|  |  |  |  |  |  |
| 0.40 | 0.55 | 1.15 |  | 0.59 0.45, 0.77 | -1 |
| 0.45 | 0.50 | 1.15 |  | 0.59 0.45, 0.77 | -1 |
| 0.50 | 0.45 | 1.15 |  | 0.58 0.45, 0.75 | 0 |
|  |  |  |  |  |  |
| 0.40 | 0.55 | 1.20 |  | 0.60 0.46, 0.78 | -3 |
| 0.45 | 0.50 | 1.20 |  | 0.59 0.45, 0.77 | -1 |
| 0.50 | 0.45 | 1.20 |  | 0.58 0.45, 0.75 | 0 |
|  |  |  |  |  |  |
| Regular contact with any pets in early childhood | | |  |  |  |
|  |  |  |  |  |  |
|  |  |  | Adjusted^3^ | 0.90 0.84, 0.96 | |
|  |  |  | Crude | 0.89 0.84, 0.95 | |
|  |  |  | External adjusted^4,5^ | |  |
| 0.50 | 0.45 | 1.10 |  | 0.89 0.84, 0.95 | 0 |
| 0.55 | 0.40 | 1.10 |  | 0.88 0.83, 0.94 | 1 |
| 0.60 | 0.35 | 1.10 |  | 0.87 0.82, 0.92 | 2 |
|  |  |  |  |  |  |
| 0.50 | 0.45 | 1.15 |  | 0.89 0.84, 0.95 | 1 |
| 0.55 | 0.40 | 1.15 |  | 0.87 0.82, 0.92 | 2 |
| 0.60 | 0.35 | 1.15 |  | 0.86 0.81, 0.91 | 4 |
|  |  |  |  |  |  |
| 0.50 | 0.45 | 1.20 |  | 0.88 0.83, 0.94 | 1 |
| 0.55 | 0.40 | 1.20 |  | 0.87 0.82, 0.92 | 3 |
| 0.60 | 0.35 | 1.20 |  | 0.85 0.80, 0.90 | 5 |

^1^ Based on paternal smoking in a sample of CLIC studies

^2^ Based on published estimates

^3^ Adjusted for child’s age at reference date, child’s sex, maternal age at child’s birth, maternal educational level and study of origin

^4^ OR calculated using the Episensi procedure in Stata command (Orsini N et al 2008)

^5^ 95%CI calculated using the following formula: exp(ln(external adjusted OR) +/- 1.96* stand error(ln (external adjusted OR))
