# Supplementary material for: Living on a farm, contact with farm animals and pets, and childhood acute lymphoblastic leukemia: pooled and meta‐analyses from the Childhood Leukemia International Consortium
Source: Cancer Med. 2018 Apr 16;7(6):2665–81. doi: 10.1002/cam4.1466 (PMC6010788; doi:10.1002/cam4.1466)
Supplement: Supplementary file 8 — Appendix S1. Acknowledgements and Funding by study. [file CAM4-7-2665-s008.doc]

Supporting information

Acknowledgements and Funding by study

**Australia, AUS_ALL**

***Research Investigators****:* Bruce Armstrong (Sydney School of Public Health), Elizabeth Milne (TICHR), Frank van Bockxmeer (Royal Perth Hospital), Michelle Haber (Children’s Cancer Institute Australia), Rodney Scott (University of Newcastle), John Attia (University of Newcastle), Murray Norris (Children’s Cancer Institute Australia), Carol Bower (TICHR), Nicholas de Klerk (TICHR), Lin Fritschi (WA Institute for Medical Research, WAIMR), Ursula Kees (TICHR), Margaret Miller (Edith Cowan University), Judith Thompson (WA Cancer Registry), Helen Bailey (TICHR). ***Clinical Investigators***: Frank Alvaro (John Hunter Hospital, Newcastle); Catherine Cole (Princess Margaret Hospital for Children, Perth); Luciano Dalla Pozza (Children’s Hospital at Westmead, Sydney); John Daubenton (Royal Hobart Hospital, Hobart); Peter Downie (Monash Medical Centre, Melbourne); Liane Lockwood, (Royal Children’s Hospital, Brisbane); Maria Kirby (Women’s and Children’s Hospital, Adelaide); Glenn Marshall (Sydney Children’s Hospital, Sydney); Elizabeth Smibert (Royal Children’s Hospital, Melbourne); Ram Suppiah, (previously Mater Children’s Hospital, Brisbane). ***Funding***: Australian National Health and Medical Research Council (Grant ID 254539).

**Brazil, BRA_SAOP**

***Main support collaborators***: Monica La Porte Teixeira, Rosa Maria Vieira Freitas (Fundacão Sistema Estadual de Ana´ lise de Dados, SEADE), Aparecida Natalia Rodrigues, Mônica Magalhães Leite, Rosemairy Inamine and Anderson da Costa. ***Funding***: Brazilian Electricity Regulatory Agency (0390-041/2004 ANNEL).

**Canada, CA_QCLS**

***Research investigator***: Claire Infante-Rivard. ***Main support collaborators***: Alexandre Cusson, Marcelle Petitclerc and Denyse Hamer. ***Funding***: The National Cancer Institute of Canada (Grant numbers: #014113, #010735-CERN #RFA0405); The Medical Research Council of Canada (Grant number: MOP 37951), The Fonds de la recherche en santé du Québec (Grant number: #981141); The Bureau of Chronic Disease Epidemiology, Canada; Health and Welfare Canada; The Leukemia Research Fund of Canada; and the National Health and Research Development Program, Ottawa.

**Costa Rica, CR_CRCLS**

***Research investigators:*** Ana M Mora (former Catharina Wesseling). ***Funding***: Research Department of the Swedish International Development Cooperation Agency (Sida/SAREC); National Cancer Institute, United States; and Universidad Nacional, Costa Rica

**France, FR_ADELE, FR_ELECTRE, FR_ESCALE and FR_ESTELLE**

***Research investigator****:* Jacqueline Clavel.***The Société Française de lutte contre les Cancers de l’Enfant et de l’Adolescent (SFCE) principal investigators***: André Baruchel (Hôpital Saint-Louis/Hôpital Robert Debré, Paris), Claire Berger (Centre Hospitalier Universitaire, Saint-Etienne), Christophe Bergeron (Centre Léon Bérard, Lyon), Jean-Louis Bernard (Hôpital La Timone, Marseille), Yves Bertrand (Hôpital Debrousse, Lyon), Pierre Bordigoni (Centre Hospitalier Universitaire, Nancy), Patrick Boutard (Centre Hospitalier Régional Universitaire, Caen), Gérard Couillault (Hôpital d’Enfants, Dijon), Christophe Piguet (Centre Hospitalier Régional Universitaire, Limoges), Anne-Sophie Defachelles (Centre Oscar Lambret, Lille), François Demeocq (Hôpital Hôtel-Dieu, Clermont-Ferrand), Alain Fischer (Hôpital des Enfants Malades, Paris), Virginie Gandemer (Centre Hospitalier Universitaire – Hôpital Sud, Rennes), Dominique Valteau-Couanet (Institut Gustave Roussy, Villejuif), Jean-Pierre Lamagnere (Centre Gatien de Clocheville, Tours), Françoise Lapierre (Centre Hospitalier Universitaire Jean Bernard, Poitiers), Guy Leverger (Hôpital Armand-Trousseau, Paris), Patrick Lutz (Hôpital de Hautepierre, Strasbourg), Geneviève Margueritte (Hôpital Arnaud de Villeneuve, Montpellier), Françoise Mechinaud (Hôpital Mère et Enfants, Nantes), Gérard Michel (Hôpital La Timone, Marseille), Frédéric Millot (Centre Hospitalier Universitaire Jean Bernard, Poitiers), Martine Münzer (American Memorial Hospital, Reims), Brigitte Nelken (Hôpital Jeanne de Flandre, Lille), Hélène Pacquement (Institut Curie, Paris), Brigitte Pautard (Centre Hospitalier Universitaire, Amiens), Stéphane Ducassou (Hôpital Pellegrin Tripode, Bordeaux), Alain Pierre-Kahn (Hôpital Enfants Malades, Paris), Emmanuel Plouvier (Centre Hospitalier Régional, Besançon), Xavier Rialland (Centre Hospitalier Universitaire, Angers), Alain Robert (Hôpital des Enfants, Toulouse), Hervé Rubie (Hôpital des Enfants, Toulouse), Stéphanie Haouy (Hôpital Arnaud de Villeneuve, Montpellier), Christine Soler (Fondation Lenval, Nice), and Jean-Pierre Vannier (Hôpital Charles Nicolle, Rouen). ***Funding****:* INSERM, the French Ministère de l’Environnement, the Agence Nationale de la Recherche (ANR) (Grant id : ANR-10-COHO-0009), the Association pour la Recherche sur le Cancer (ARC), the Agence Française de Sécurité Sanitaire des Produits de Santé (AFSSAPS), the Agence Française de Sécurité Sanitaire de l’Environnement et du Travail (AFSSET), the Agence Nationale de Sécurité Sanitaire de l’alimentation, de l’Environnement et du Travail (PNREST Anses, Cancer TMOI AVIESAN, 2013/1/248), the association Cent pour sang la vie, the association Enfants et Santé, the Cancéropôle Ile-de-France, the Fondation de France, the Fondation Jeanne Liot, the Fondation pour la Recherche Médicale, the Fondation Weisbrem-Berenson, the Institut National du Cancer (INCa), the Ligue Contre le Cancer du Val de Marne, the Ligue Nationale Contre le Cancer (LNCC), the Institut Electicité Santé.

**Greece, GR_NARECHEM**

***Research investigators****:* Eleni T. Petridou, Nick Dessypris.***Clinical investigators***: Margarita Baka MD: Department of Pediatric Hematology –Oncology, “Pan.&Agl. Kyriakou” Children’s Hospital, Athens, Greece, Thivon & Levadeias, Goudi; Maria Moschovi  MD: Hematology-Oncology Unit, First Department of Pediatrics, Athens University Medical School, “Aghia Sophia” General Children's Hospital, Athens, Greece, Thivon & Papadiamantopoulou, Goudi, 11527 Athens, Greece; Sophia Polychronopoulou  MD: Department of Pediatric Hematology-Oncology, “Aghia Sophia” General Children's Hospital, Athens, Greece, Thivon & Papadiamantopoulou, Goudi, 11527 Athens, Greece; Emmanuel Hatzipantelis MD, PhD: Pediatric Hematology Oncology Unit, 2nd Pediatric Department of Aristotle University, AHEPA General Hospital, Thessaloniki, Greece, 1 St. Kyriakidi, 54636 Thessaloniki, Greece; Ioanna Fragandrea MD: Pediatric Oncology Department, Hippokration Hospital, Thessaloniki, Greece ; Eftychia Stiakaki MD: Department of Pediatric Hematology-Oncology, University Hospital of Heraklion, Heraklion, Greece; Nick Dessypris, MSc, PhD and Evanthia Bouka, MPH: Department of Hygiene, Epidemiology and Medical Statistics, Athens University Medical School, 11527 Athens, Greece; Ioannis Matsoukis MD: Department of Hygiene, Epidemiology and Medical Statistics, Athens University Medical School, 11527 Athens, Greece; Maria Kourti MD : Department of Pediatric Hematology and Oncology, Hippokration Hospital, Thessaloníki, Greece. ***Funding***: National and Kapodistrian University, Athens, Greece.

**Italy, IT_SETIL**

***Working Group***: Corrado Magnani and Alessandra Ranucci (Cancer Epidemiology Unit, CPO Piedmont Novara); Lucia Miligi, Alessandra Benvenuti, Patrizia Legittimo and Angela Veraldi (Occupational and Environmental Unit ,ISPO, Firenze); Antonio Acquaviva (AOU Siena); Maurizio Aricò, Alma Lippi and Gabriella Bernini (AOU Meyer, Firenze); Giorgio Assennato (ARPA, Bari); Stefania Varotto and Paola Zambon (Università di Padova); Pierfranco Biddau and Roberto Targhetta (Ospedale Microcitemico, Cagliari); Luigi Bisanti and Giuseppe Sampietro (ASL di Milano); Francesco Bochicchio, Susanna Lagorio, Cristina Nuccetelli, Alessandro Polichetti and Serena Risica, (ISS, Roma); Santina Cannizzaro and Lorenzo Gafà (LILT, Ragusa); Egidio Celentano (ARSan, Napoli); Pierluigi Cocco (Università di Cagliari); Marina Cuttini (IRCCS Burlo Garofolo, Trieste); Francesco Forastiere,Ursula Kirchmayer and Paola Michelozzi (Dipartimento Epidemiologia Regione Lazio, Roma); Erni Guarino (INT Napoli); Riccardo Haupt (Istituto Giannina Gaslini, Genova); Franco Locatelli (Università di Pavia and AO Bambin Gesù, Roma); Lia Lidia Luzzatto (ASL 1 , Torino); Giuseppe Masera (Università Milano Bicocca, Monza); Pia Massaglia (Università di Torino); Stefano Mattioli and Andrea Pession (Università di Bologna); Domenico Franco Merlo and Vittorio Bocchini (IST, Genova); Liliana Minelli and Manuela Chiavarini (Università degli Studi di Perugia);Margherita Nardi (AOU Pisa); Paola Mosciatti and Franco Pannelli (Università di Camerino);Vincenzo Poggi (AORN Santobono – Pausilipon, Napoli);Alessandro Pulsoni (Sapienza University, Roma); Carmelo Rizzari (AO San Gerardo, Monza); Roberto Rondelli (Policlinico S.Orsola, Bologna); Gino Schilirò (Università di Catania); Alberto Salvan (IASI-CNR, Roma); Maria Valeria Torregrossa and Rosaria Maria Valenti, (Università degli Studi di Palermo);Alessandra Greco, Gian Luca DeSalvo and Daniele Monetti (IOV-IRCCS, Padova); Claudia Galassi (San Giovanni Battista Hospital, Torino); Veronica Casotto (IRCCS Burlo Garofolo, Trieste); Gigliola de Nichilo (ASL BT, SPRESAL Barletta); Alberto Cappelli, (Accademia dei Georgofili, Florence). ***Funding****:* Italian Association on Research on Cancer (AIRC); Ministry for Instruction, University and Research (MIUR); Ministry of Health; Ministry of Labour, Piedmont Region.

**New Zealand, NZ_NZCCS**

***Research investigator****:* JD Dockerty, PG Herbison, DCG Skegg and JM Elwood (University of Otago). ***Funding****:* the Health Research Council of NZ, the NZ Lottery Grants Board, the Otago Medical School (Faculty Bequest Funds), the Cancer Society of NZ, the Otago Medical Research Foundation, and the A.B. de Lautour Charitable Trust.

**United States, US_COG15**

The E14 and E15 cohorts of the Children’s Oncology Group was identified by CCG (Children’s Cancer Group) principle and affiliate member institutions. Further information can be found on the web-site: http://www.curesearch.org/.

**United States, US_NCCLS**

***Research investigators:*** Catherine Metayer***. Clinical investigators***: J. Ducore (University of California Davis Medical Center); M. Loh and K. Matthay (University of California San Francisco), V. Crouse (Children's Hospital of Central California), G. Dahl (Lucile Packard Children's Hospital), J. Feusner (Children's Hospital Oakland), K. Jolly and V. Kiley (Kaiser Permanente Roseville), C. Russo, A. Wong, and D. Taggar (Kaiser Permanente Santa Clara), K. Leung (Kaiser Permanente San Francisco), and D. Kronish and S. Month (Kaiser Permanente Oakland). ***Funding***: National Institutes of Health (NIH), USA (grants P01 ES018172, R01 ES09137, and P42-ES04705), Environmental Protection Agency (USEPA), USA (grant RD83451101), and the CHILDREN with CANCER (CwC), UK (former Children with Leukaemia) for data collection.

The content is solely the responsibility of the authors and does not necessarily represent the official views of the NIH, USEPA, or the CwC.

**Childhood Leukemia International Consortium.**

***Funding****:* National Cancer Institute, NCI, USA (grant R03CA132172), National Institute of Environmental Health Sciences, NIEHS, USA (grants P01 ES018172 and R13 ES021145-01), the Environmental Protection Agency, EPA, USEPA, USA (grant RD83451101), and the Children with Cancer, CwC, UK (Award No. 2010/097).
