# Supplementary material for: Living on a farm, contact with farm animals and pets, and childhood acute lymphoblastic leukemia: pooled and meta‐analyses from the Childhood Leukemia International Consortium
Source: Cancer Med. 2018 Apr 16;7(6):2665–81. doi: 10.1002/cam4.1466 (PMC6010788; doi:10.1002/cam4.1466)
Supplement: Supplementary file 9 [file CAM4-7-2665-s009.doc]

**Supporting Information : Web Figures Legend**

**Web Figure 1.** Association between Acute Lymphoblastic Leukemia and Contact with Cattle in the First Year of Life (yes *vs* no), Restricted to Children Aged ≥1 Year, Meta-Analysis of 7 studies (1990-2013), Childhood Leukemia International Consortium.

Abbreviations: ALL, acute lymphoblastic leukemia; CI, confidence interval; CR_CRLS, Costa Rican Childhood Leukemia Study (Costa Rica); FR_ADELE, Adele study (France); FR_ELECTRE, Electre study (France); FR_ESCALE, Epidemiological study on childhood cancer and leukemia (France); FR_ESTELLE, Epidemiologic Study on Childhood Cancer, Leukemia and lymphoma (France); GR_NARECHEM, Nationwide registration for childhood haematological malignancies (Greece); NZ_NZCCS, New Zealand childhood cancer study (New Zealand); OR, odds ratio; Pe_ALL, prevalence of exposure among acute lymphoblastic leukemia cases; Pe_cont, prevalence of exposure among controls.

Studies are ordered by increasing study period. Study-specific Odds ratios and 95% confidence interval were estimated by conditional (GR_NARECHEM, NZ_NZCCS) or unconditional (CR_CRLS, FR_ADELE, FR_ELECTRE, FR_ESCALE, FR_ESTELLE) logistic models, adjusted for child’s age at reference date, sex, maternal educational level (FR_ELECTRE, GR_NARECHEM, NZ_NZCCS), ethnicity (FR_ADELE, NZ_NZCCS), region or center of recruitment (FR_ADELE), region or state of residence (FR_ELECTRE), "urban/rural" status of the place of residence (GR_NARECHEM, FR_ESTELLE), parental professional category (CR_CRLS, FR_ELECTRE, FR_ESCALE, FR_ESTELLE, GR_NARECHEM), maternal age at child’s birth (FR_ELECTRE, FR_ESCALE, FR_ESTELLE).

**Web Figure 2.** Association between Acute Lymphoblastic Leukemia and Contact with Pigs in the First Year of Life (yes *vs* no), Restricted to Children Aged ≥1 Year, Meta-Analysis of 7 studies (1990-2013), Childhood Leukemia International Consortium.

Abbreviations: ALL, acute lymphoblastic leukemia; CI, confidence interval; CR_CRLS, Costa Rican Childhood Leukemia Study (Costa Rica); FR_ADELE, Adele study (France); FR_ELECTRE, Electre study (France); FR_ESCALE, Epidemiological study on childhood cancer and leukemia (France); FR_ESTELLE, Epidemiologic Study on Childhood Cancer, Leukemia and lymphoma (France); GR_NARECHEM, Nationwide registration for childhood haematological malignancies (Greece); NZ_NZCCS, New Zealand childhood cancer study (New Zealand); OR, odds ratio; Pe_ALL, prevalence of exposure among acute lymphoblastic leukemia cases; Pe_cont, prevalence of exposure among controls.

Studies are ordered by increasing study period. Study-specific Odds ratios and 95% confidence interval were estimated by conditional (GR_NARECHEM, NZ_NZCCS) or unconditional (CR_CRLS, FR_ADELE, FR_ELECTRE, FR_ESCALE, FR_ESTELLE) logistic models, adjusted for child’s age at reference date, sex, maternal educational level (FR_ELECTRE, GR_NARECHEM, NZ_NZCCS), ethnicity (FR_ADELE, NZ_NZCCS), region or center of recruitment (FR_ADELE), region or state of residence (FR_ELECTRE), "urban/rural" status of the place of residence (GR_NARECHEM, FR_ESTELLE), parental professional category (CR_CRLS, FR_ELECTRE, FR_ESCALE, FR_ESTELLE, GR_NARECHEM), maternal age at child’s birth (FR_ELECTRE, FR_ESCALE, FR_ESTELLE).

**Web Figure 3.** Association between Acute Lymphoblastic Leukemia and Contact with Sheep in the First Year of Life (yes *vs* no), Restricted to Children Aged ≥1 Year, Meta-Analysis of 6 studies (1990-2013), Childhood Leukemia International Consortium.

Abbreviations: ALL, acute lymphoblastic leukemia; CI, confidence interval; FR_ADELE, Adele study (France); FR_ELECTRE, Electre study (France); FR_ESCALE, Epidemiological study on childhood cancer and leukemia (France); FR_ESTELLE, Epidemiologic Study on Childhood Cancer, Leukemia and lymphoma (France); GR_NARECHEM, Nationwide registration for childhood haematological malignancies (Greece); NZ_NZCCS, New Zealand childhood cancer study (New Zealand); OR, odds ratio; Pe_ALL, prevalence of exposure among acute lymphoblastic leukemia cases; Pe_cont, prevalence of exposure among controls.

Studies are ordered by increasing study period. Study-specific Odds ratios and 95% confidence interval were estimated by conditional (GR_NARECHEM, NZ_NZCCS) or unconditional (FR_ADELE, FR_ELECTRE, FR_ESCALE, FR_ESTELLE) logistic models, adjusted for child’s age at reference date, sex, maternal educational level (FR_ELECTRE, GR_NARECHEM, NZ_NZCCS), ethnicity (FR_ADELE, NZ_NZCCS), region or center of recruitment (FR_ADELE), region or state of residence (FR_ELECTRE), "urban/rural" status of the place of residence (GR_NARECHEM, FR_ESTELLE), parental professional category (FR_ELECTRE, FR_ESCALE, FR_ESTELLE, GR_NARECHEM), maternal age at child’s birth (FR_ELECTRE, FR_ESCALE, FR_ESTELLE).

**Web Figure 4.** Association between Acute Lymphoblastic Leukemia and Contact with Poultry in the First Year of Life (yes *vs* no), Restricted to Children Aged ≥1 Year, Meta-Analysis of 6 studies (1990-2013), Childhood Leukemia International Consortium.

Abbreviations: ALL, acute lymphoblastic leukemia; CI, confidence interval; FR_ADELE, Adele study (France); FR_ELECTRE, Electre study (France); FR_ESCALE, Epidemiological study on childhood cancer and leukemia (France); FR_ESTELLE, Epidemiologic Study on Childhood Cancer, Leukemia and lymphoma (France); GR_NARECHEM, Nationwide registration for childhood haematological malignancies (Greece); NZ_NZCCS, New Zealand childhood cancer study (New Zealand); OR, odds ratio; Pe_ALL, prevalence of exposure among acute lymphoblastic leukemia cases; Pe_cont, prevalence of exposure among controls.

Studies are ordered by increasing study period. Study-specific Odds ratios and 95% confidence interval were estimated by conditional (GR_NARECHEM, NZ_NZCCS) or unconditional (FR_ADELE, FR_ELECTRE, FR_ESCALE, FR_ESTELLE) logistic models, adjusted for child’s age at reference date, sex, maternal educational level (FR_ELECTRE, GR_NARECHEM, NZ_NZCCS), ethnicity (FR_ADELE, NZ_NZCCS), region or center of recruitment (FR_ADELE), region or state of residence (FR_ELECTRE), "urban/rural" status of the place of residence (GR_NARECHEM, FR_ESTELLE), parental professional category (FR_ELECTRE, FR_ESCALE, FR_ESTELLE, GR_NARECHEM), maternal age at child’s birth (FR_ELECTRE, FR_ESCALE, FR_ESTELLE).

**Web Figure 5.** Association between Acute Lymphoblastic Leukemia and Contact with Dogs in the First Year of Life (yes *vs* no), Restricted to Children Aged ≥1 year, Meta-Analysis of 12 studies (1980-2013), Childhood Leukemia International Consortium.

Abbreviations: ALL, acute lymphoblastic leukemia, BRA_SAOP, State of Sao Paulo Childhood acute lymphoblastic leukemia study (Brazil); CA_QCLS, Quebec childhood leukemia study (Canada); CI, confidence interval; CR_CRLS, Costa Rican Childhood Leukemia Study (Costa Rica); FR_ADELE, Adele study (France); FR_ELECTRE, Electre study (France); FR_ESCALE, Epidemiological study on childhood cancer and leukemia (France); FR_ESTELLE, Epidemiologic Study on Childhood Cancer, Leukemia and lymphoma (France); GR_NARECHEM, Nationwide registration for childhood haematological malignancies (Greece); IT_SETIL, Study on the etiology of childhood lymphohematopoietic malignancies (Italy); NZ_NZCCS, New Zealand childhood cancer study (New Zealand); OR, odds ratio; Pe_ALL, prevalence of exposure among acute lymphoblastic leukemia cases; Pe_cont, prevalence of exposure among controls; US_COG15, Children’s oncology group study (US); US_NCCLS, Northern California childhood leukemia study (US).

Studies are ordered by increasing study period. Study-specific Odds ratios and 95% confidence interval were estimated by conditional (CA_QCLS, GR_NARECHEM, NZ_NZCCS, US_COG15, US_NCCLS) or unconditional (BRA_SAOP, CR_CRLS, FR_ADELE, FR_ELECTRE, FR_ESCALE, FR_ESTELLE, IT_SETIL) logistic models, adjusted for child’s age at reference date, sex, maternal educational level (BRA_SAOP, FR_ELECTRE, GR_NARECHEM, IT_SETIL, NZ_NZCCS, US_COG15, US_NCCLS), ethnicity (FR_ADELE, NZ_NZCCS, US_COG15), region or center of recruitment (FR_ADELE), region or state of residence (FR_ELECTRE), "urban/rural" status of the place of residence (GR_NARECHEM, FR_ESTELLE), parental professional category (BRA_SAOP, CR_CRLS, FR_ELECTRE, FR_ESCALE, FR_ESTELLE, GR_NARECHEM), household income (US_COG15, US_NCCLS), maternal age at child’s birth (BRA_SAOP, CA_QCLS, FR_ELECTRE, FR_ESCALE, FR_ESTELLE, US_NCCLS).

**Web Figure 6.** Association between Acute Lymphoblastic Leukemia and Contact with Cats in the First Year of Life (yes *vs* no), Restricted to Children Aged ≥1 year, Meta-Analysis of 12 studies (1980-2013), Childhood Leukemia International Consortium.

Abbreviations: ALL, acute lymphoblastic leukemia, BRA_SAOP, State of Sao Paulo Childhood acute lymphoblastic leukemia study (Brazil); CA_QCLS, Quebec childhood leukemia study (Canada); CI, confidence interval; CR_CRLS, Costa Rican Childhood Leukemia Study (Costa Rica); FR_ADELE, Adele study (France); FR_ELECTRE, Electre study (France); FR_ESCALE, Epidemiological study on childhood cancer and leukemia (France); FR_ESTELLE, Epidemiologic Study on Childhood Cancer, Leukemia and lymphoma (France); GR_NARECHEM, Nationwide registration for childhood haematological malignancies (Greece); IT_SETIL, Study on the etiology of childhood lymphohematopoietic malignancies (Italy); NZ_NZCCS, New Zealand childhood cancer study (New Zealand); OR, odds ratio; Pe_ALL, prevalence of exposure among acute lymphoblastic leukemia cases; Pe_cont, prevalence of exposure among controls; US_COG15, Children’s oncology group study (US); US_NCCLS, Northern California childhood leukemia study (US).

Studies are ordered by increasing study period. Study-specific Odds ratios and 95% confidence interval were estimated by conditional (CA_QCLS, GR_NARECHEM, NZ_NZCCS, US_COG15, US_NCCLS) or unconditional (BRA_SAOP, CR_CRLS, FR_ADELE, FR_ELECTRE, FR_ESCALE, FR_ESTELLE, IT_SETIL) logistic models, adjusted for child’s age at reference date, sex, maternal educational level (BRA_SAOP, FR_ELECTRE, GR_NARECHEM, IT_SETIL, NZ_NZCCS, US_COG15, US_NCCLS), ethnicity (FR_ADELE, NZ_NZCCS, US_COG15), region or center of recruitment (FR_ADELE), region or state of residence (FR_ELECTRE), "urban/rural" status of the place of residence (GR_NARECHEM, FR_ESTELLE), parental professional category (BRA_SAOP, CR_CRLS, FR_ELECTRE, FR_ESCALE, FR_ESTELLE, GR_NARECHEM), household income (US_COG15, US_NCCLS), maternal age at child’s birth (BRA_SAOP, CA_QCLS, FR_ELECTRE, FR_ESCALE, FR_ESTELLE, US_NCCLS).
